# Longitudinal and Multi-Kingdom Gut Microbiome Alterations in a Mouse Model of Alzheimer’s Disease

**DOI:** 10.3390/ijms252111472

**Published:** 2024-10-25

**Authors:** Tao Zhang, Chunyan Zhao, Na Li, Qiuwen He, Guangqi Gao, Zhihong Sun

**Affiliations:** 1 Key Laboratory of Dairy Biotechnology and Engineering, Ministry of Education, Inner Mongolia Agricultural University, Hohhot 010018, China; 18736000538@163.com (T.Z.); 13948539834@163.com (C.Z.); 18004899551@163.com (N.L.); heidyww@163.com (Q.H.); 2 Key Laboratory of Dairy Products Processing, Ministry of Agriculture and Rural Affairs, Inner Mongolia Agricultural University, Hohhot 010018, China; 3 Inner Mongolia Key Laboratory of Dairy Biotechnology and Engineering, Inner Mongolia Agricultural University, Hohhot 010018, China; 4 Collaborative Innovative Center for Lactic Acid Bacteria and Fermented Dairy Products, Ministry of Education, Inner Mongolia Agricultural University, Hohhot 010018, China

**Keywords:** Alzheimer’s disease, gut microbiome, bacteriome, mycobiome, archaeome, virome

## Abstract

Gut microbial dysbiosis, especially bacteriome, has been implicated in Alzheimer’s disease (AD). However, nonbacterial members of the gut microbiome in AD, such as the mycobiome, archaeome, and virome, are unexplored. Here, we perform higher-resolution shotgun metagenomic sequencing on fecal samples collected longitudinally from a mouse model of AD to investigate longitudinal and multi-kingdom gut microbiome profiling. Shotgun metagenomic sequencing of fecal samples from AD mice and healthy mice returns 41,222 bacterial, 414 fungal, 1836 archaeal, and 1916 viral species across all time points. The ecological network pattern of the gut microbiome in AD mice is characterized by more complex bacterial–bacterial interactions and fungal–fungal interactions, as well as simpler archaeal–archaeal interactions and viral–viral interactions. The development of AD is accompanied by multi-kingdom shifts in the gut microbiome composition, as evidenced by the identification of 1177 differential bacterial, 84 differential fungal, 59 differential archaeal, and 10 differential viral species between healthy and AD mice across all time points. In addition, the functional potential of the gut microbiome is partially altered in the development of AD. Collectively, our findings uncover longitudinal and multi-kingdom gut microbiome alterations in AD and provide a motivation for considering microbiome-based therapeutics during the prevention and treatment of AD.

## 1. Introduction

Alzheimer’s disease (AD) is the most common neurodegenerative disorder in the elderly; it is characterized by a progressive decline in cognitive abilities with neuropathological hallmarks of the accumulation of extracellular amyloid-beta (Aβ) plaques and intracellular tau neurofibrillary tangles [1]. Although these two features are biologically responsible for the pathogenesis of AD, there are fewer therapeutic regimens that directly target the biological underpinnings of the condition. In fact, of these limited treatment options, only lecanemab has been fully approved by the U.S. Food and Drug Administration for the treatment of early AD after an 18-month, multicenter, double-blind, phase 3 trial [2], opening up a new era of disease-modifying therapies for the disease. However, along with the excitement, the clinical benefits produced by the Aβ-directed antibody are simultaneously accompanied by adverse events such as localized brain swelling or hemorrhages [2]. Concerted efforts to develop new therapeutic approaches without apparent side effects would therefore tremendously expand AD therapy armamentarium.

Evidence is accumulating relating to the presence of a long-distance connection between the gut microbiome and AD. These preliminary associations have been corroborated by human and animal observational studies that have shown alterations in the gut microbiome using high-throughput microbial genomic sequencing [3]. Subsequently, the translation of these associations into causality began through fecal microbiota transplantation experiments designed to confirm that the gut microbiome serves as a key driver of AD [4,5,6]. Together, these data suggest that the gut microbiome may be involved in the onset and progression of AD. An important previous study showing that gut-derived *Faecalibacterium prausnitzii* can improve Aβ-induced cognitive impairment in mice [7] not only strengthens the aforementioned concept, but also introduces the significance of identifying specific gut microbes associated with AD. The comprehensive profiling of gut microbiota in AD might therefore pave the way for novel treatment options.

Although efforts have been made to characterize the gut microbiome in the setting of AD, there are still some shortcomings. First, 16S ribosomal RNA gene sequencing has been widely used in animal and human studies of AD to investigate the impact of gut microbiome on disease [3]; however, this approach cannot achieve microbial species-level or strain-level resolution. Second, nearly all of the work in this field to date has primarily focused on intestinal bacteria while overlooking the contribution of nonbacterial members of the gut microbiome to human health and disease, such as mycobiome [8,9], virome [10], and archaeome [11]. Third, there is a lack of knowledge about the longitudinal gut microbiome configuration that precedes the onset of AD pathology.

Thus, to address these constraints, in the present study, we performed higher-resolution shotgun metagenomic sequencing on fecal samples at distinct time points obtained from APP/PS1 mice, the most commonly used mouse model of AD, to comprehensively characterize the landscape of the gut microbiome and its interactions. Our work would provide readers not only with a comprehensive blueprint of the gut microbiome of AD, but also with a rationale for microbial therapeutics for AD.

## 2. Results

### 2.1. APP/PS1 Mice Can Mimic the Pathology of AD

**Figure 1:**
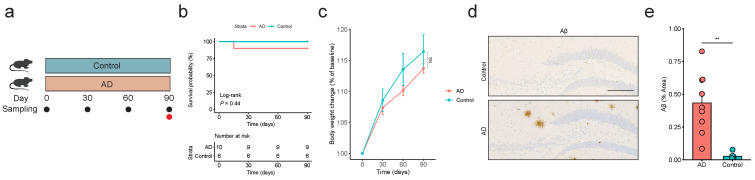
**APP/PS1 mice can mimic the pathology of Alzheimer’s disease (AD).** (**a**) Experimental design. Wild-type mice from the Control group (n = 6) and APP/PS1 mice from the AD group (n = 10) were raised conventionally for 90 days from the age of 3 months old to the experimental end point. The sampling times for fecal and brain tissue samples are denoted by black and red circles, respectively. (**b**) Survival of wild-type and APP/PS1 mice during the animal experiment. (**c**) Body weight change in mice in the Control and AD groups. (**d**) Representative immunohistochemistry images of hippocampal sections from wild-type and APP/PS1 mice stained with antibodies to amyloid-beta (Aβ). Scale bar: 200 μm. (**e**) Percentage of the area covered by Aβ staining of hippocampal sections prepared from wild-type and APP/PS1 mice. Data are represented as mean  ±  standard error of the mean (s.e.m). ** *p* < 0.01; NS, not significant. Statistical differences were determined by log-rank test (**b**) and two-sided Wilcoxon rank-sum test (**c**,**e**).

Wild-type and APP/PS1 mice were raised conventionally for 90 days from the age of 3 months old to 6 months old (Figure 1a). Given the potential emergence of deaths due to a high prevalence of seizures in APP/PS1 mice [12], we initially calculated the overall survival of the mice. As expected, one mouse in the AD group died naturally (no wounds found), which was not observed in the Control group (Figure 1b). The log-rank test showed no striking difference in terms of survival probability between the two groups (Figure 1b). In addition, mice in the AD group demonstrated less body weight change by the end of the experiment relative to controls (Figure 1c). As the deposition of Aβ plaques in the brain of APP/PS1 mice is considered the gold standard for the pathological hallmarks of AD [13], we therefore performed immunohistochemistry staining of mouse brains to detect Aβ. As expected, APP/PS1 mice displayed severe Aβ plaques in the hippocampus compared with wild-type mice (Figure 1d), which was further quantitatively supported by a significant increase in the percent of the hippocampal area covered by Aβ staining (Figure 1e). Together, these data indicate that APP/PS1 mice can reliably act as a mouse model of AD.

### 2.2. Characterization of Longitudinal and Multi-Kingdom Gut Microbiome Datasets from AD Mice

**Figure 2:**
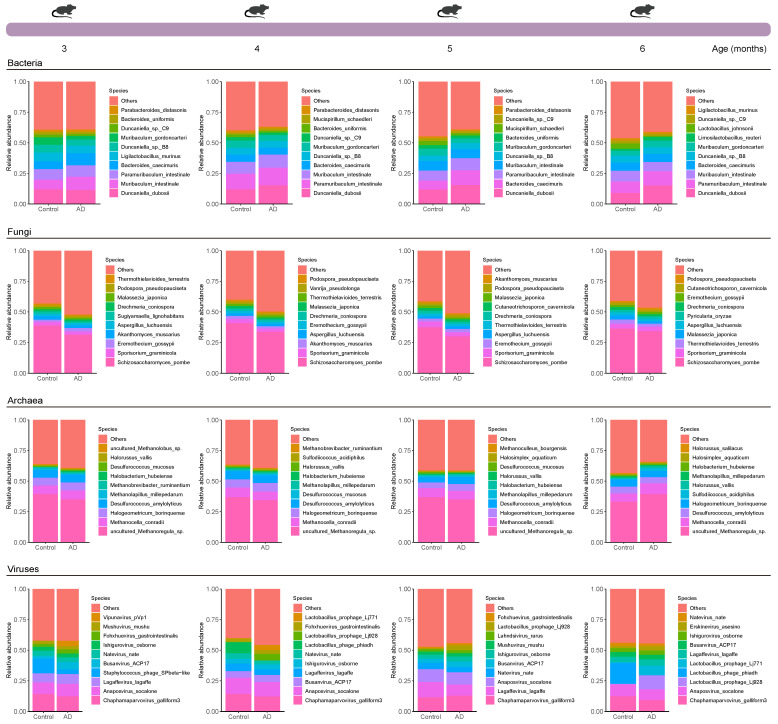
**Overview of the dominant multi-kingdom species composition in mice**. Distribution of the most abundant bacterial, fungal, archaeal, and viral species in mice from the Control and Alzheimer’s disease (AD) groups at months 3–6 based on mean relative abundance. The species ranked from 11th to last, based on mean relative abundance across all samples, were grouped as ‘Others’.

To investigate the longitudinal and multi-kingdom gut microbiome profile in mice, we performed shotgun metagenomic sequencing on DNA from 60 longitudinal fecal samples collected from nine APP/PS1 mice and six controls at four time points (3, 4, 5, and 6 months of age). We first used Kraken2 to annotate the gut bacteria of mice from the Control and AD groups, and then used Bracken to estimate their abundance, yielding 10,320, 10,296, 10,335, and 10,271 bacterial species at different time points, respectively (Supplementary Figure S1a–d). Seven phyla were identified across the two groups, including Actinomycetota, Bacillota, Bacteroidota, Campylobacterota, Deferribacterota, Pseudomonadota, and Verrucomicrobiota, with the most dominant phyla being Bacteroidota and Bacillota (Supplementary Figure S1a–d). Apart from gut bacteria, evidence is emerging that nonbacterial members in the gut may also participate in gut physiology and pathogenesis [14]. We thus annotated fungi, archaea, and viruses from fecal samples using the same tools. For fungi, we identified 103, 104, 104, and 103 species at different time points, respectively, originating from three phyla, namely Ascomycota, Basidiomycota, and Microsporidia (Supplementary Figure S2a–d). For archaea, 460, 459, 461, and 456 archaeal species were classified at different time points, respectively, grouped by the phyla Candidatus Thermoplasmatota, Euryarchaeota, Nitrososphaerota, and Thermoproteota (Supplementary Figure S3a–d). For viruses, we identified 498, 485, 473, and 460 viral species at different time points, respectively, which mainly belonged to the Uroviricota phylum (Supplementary Figure S4a–d). The dominant multi-kingdom species at various time points were further determined between the Control and AD groups (Figure 2). Furthermore, additional data per mouse at the multi-kingdom species level can be found in Supplementary Figure S5. Overall, our results establish a comprehensive longitudinal and multi-kingdom intestinal microbial landscape in relation to AD.

### 2.3. Characterization of Longitudinal and Multi-Kingdom Ecological Networks in AD Mice

**Figure 3:**
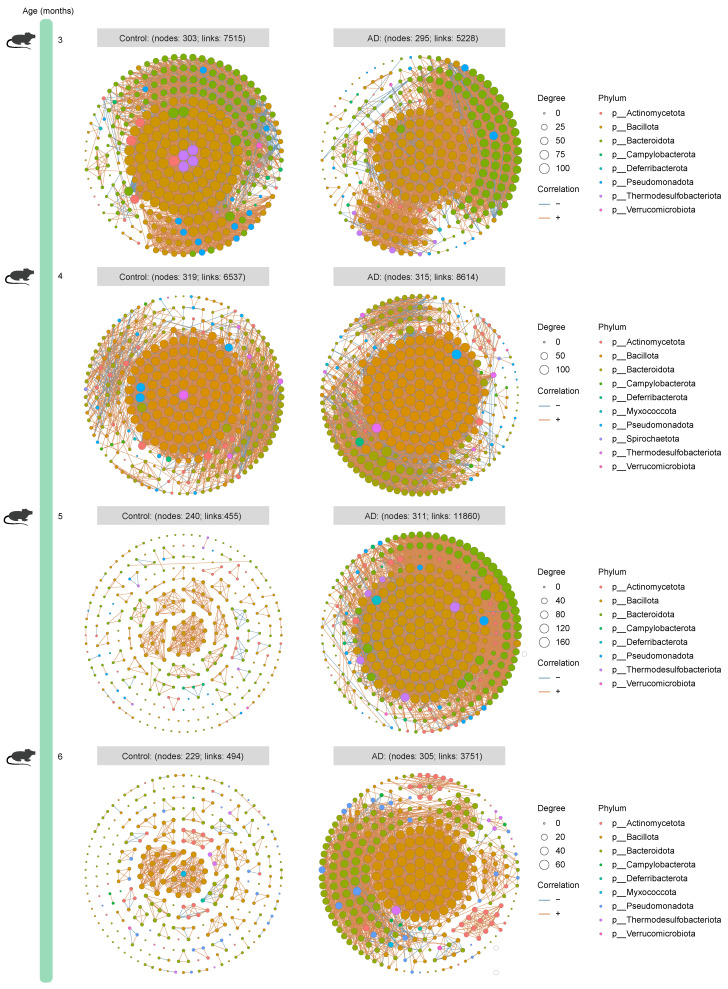
**Time-series bacterial ecological network of mice from the Control and Alzheimer’s disease (AD) groups**. The circles represent all bacterial species, and the colors of the circles represent phyla. The nodes represent partial bacterial species, which have a positive or negative correlation with other bacteria. The node size is proportional to the degree. The rust orange indicates positive correlations, whereas the sky blue indicates negative correlations. Only bacterial species with a mean relative abundance of at least 0.01% were used for network construction. Only networks with absolute values of Spearman’s correlation coefficient greater than 0.8 and *Q* values less than 0.05 were shown.

**Figure 4:**
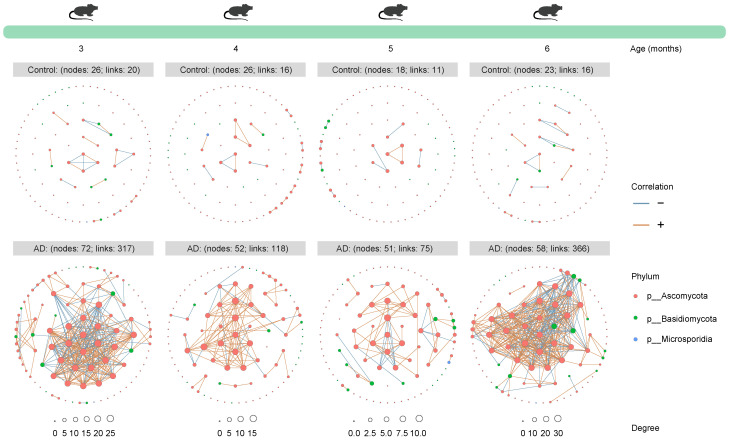
**Time-series fungal ecological network of mice from the Control and Alzheimer’s disease (AD) groups**. The circles represent all fungal species, and the colors of the circles represent phyla. The nodes represent partial fungal species, which have a positive or negative correlation with other fungi. The node size is proportional to the degree. The rust orange indicates positive correlations, whereas the sky blue indicates negative correlations. Only fungal species with a mean relative abundance of at least 0.01% were used for network construction. Only networks with absolute values of Spearman’s correlation coefficient greater than 0.8 and *Q* values less than 0.05 were shown.

**Figure 5:**
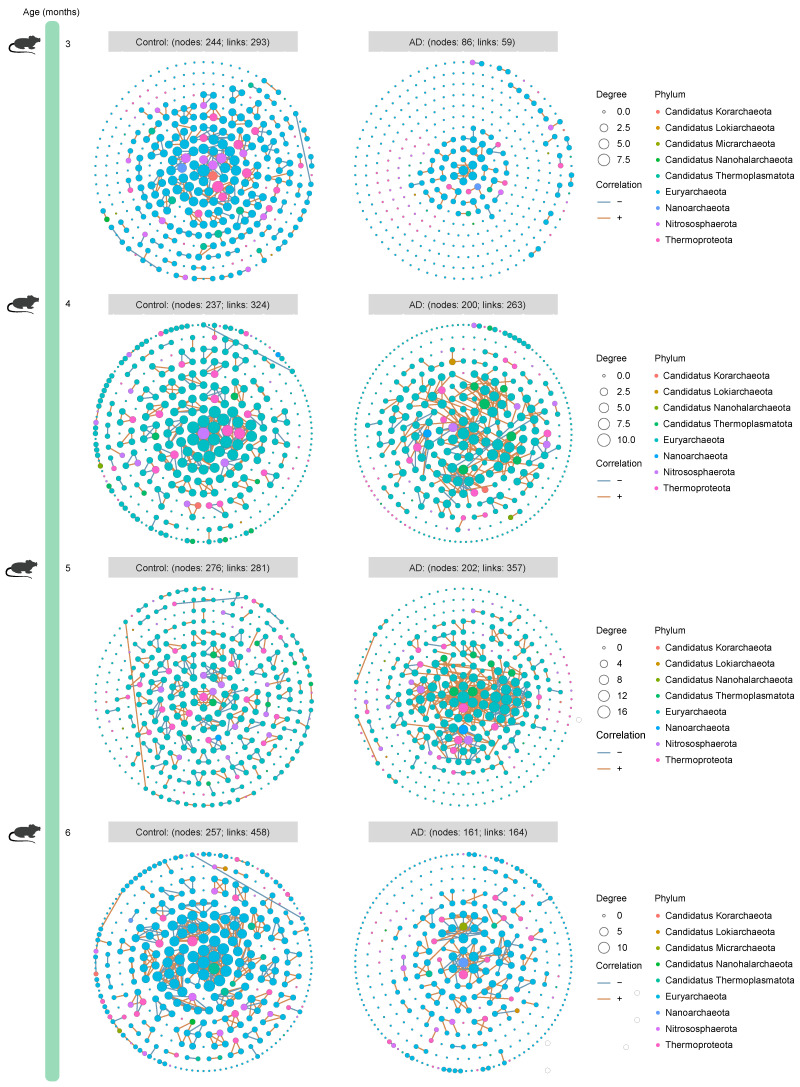
**Time-series archaeal ecological network of mice from the Control and Alzheimer’s disease (AD) groups**. The circles represent all archaeal species, and the colors of the circles represent phyla. The nodes represent partial archaeal species, which have a positive or negative correlation with other archaea. The node size is proportional to the degree. The rust orange indicates positive correlations, whereas the sky blue indicates negative correlations. Only archaeal species with a mean relative abundance of at least 0.01% were used for network construction. Only networks with absolute values of Spearman’s correlation coefficient greater than 0.8 and *Q* values less than 0.05 were shown.

**Figure 6:**
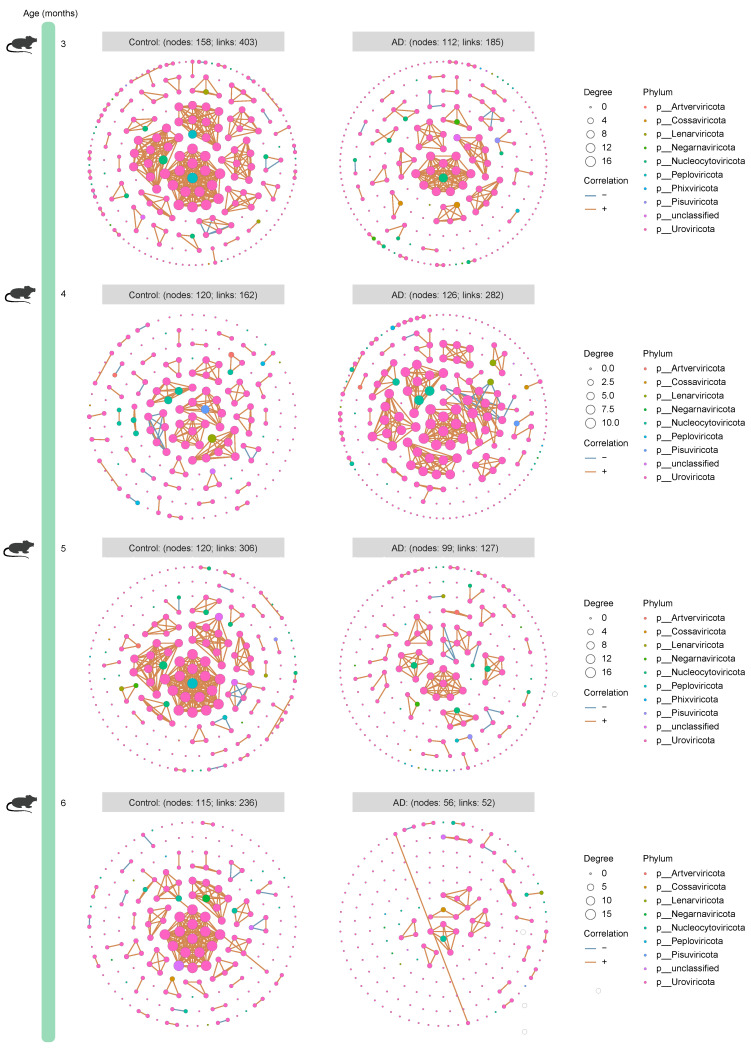
**Time-series viral ecological network of mice from the Control and Alzheimer’s disease (AD) groups**. The circles represent all viral species, and the colors of the circles represent phyla. The nodes represent partial viral species, which have a positive or negative correlation with other viruses. The node size is proportional to the degree. The rust orange indicates positive correlations, whereas the sky blue indicates negative correlations. Only viral species with a mean relative abundance of at least 0.01% were used for network construction. Only networks with absolute values of Spearman’s correlation coefficient greater than 0.8 and *Q* values less than 0.05 were shown.

To dissect the microbiota–microbiota interactions in the gut of AD mice and healthy mice, we established time-series ecological networks of multi-kingdom microbial communities based on detected species abundance. Although the bacterial ecological network of 3-month-old healthy mice (303 nodes and 7515 associations) was more complex than that of age-matched AD mice (295 nodes and 5228 associations), opposite trends were consistently observed when the mice were 4 (Control group: 319 nodes and 6537 associations; AD group: 315 nodes and 8614 associations), 5 (Control group: 240 nodes and 455 associations; AD group: 311 nodes and 11,860 associations), and 6 (Control group: 229 nodes and 494 associations; AD group: 305 nodes and 3751 associations) months old (Figure 3). Surprisingly, the number of nodes and correlations in the fungal ecological network of AD mice was higher than that of healthy mice across all time points (Figure 4), suggesting a continued complexity of the fungal ecological network as the disease progressed. The archaeal ecological network of AD mice was uncomplicated at different time points (except at 5 months of age) compared to healthy mice (Figure 5). Similarly, a more complex viral ecological network was observed only in 4-month-old AD mice (Control group: 120 nodes and 162 associations; AD group: 126 nodes and 282 associations) compared to age-matched healthy mice, while the opposite pattern was observed at the other three time points (Figure 6). Overall, these results indicate that AD mice have a more complex bacterial and fungal ecological network, and a simpler archaeal and viral ecological network.

### 2.4. Persistent Changes in the Gut Fungal and Viral Community Structure

To investigate the gut microbiota diversity between the Control and AD groups, we evaluated the alpha and beta diversity metrics at the species level annotated from shotgun metagenomic sequencing. Although no significant differences in bacterial alpha diversity were noted between the two groups at the first two time points, a lower alpha diversity (Simpson index, *p* = 0.0496 and *p* = 0.0256, respectively) was observed in 5- and 6-month-old AD mice (Supplementary Figure S6). AD mice showed a significant increase in the fungal alpha diversity compared with controls at almost all time points (Supplementary Figure S6). Archaeal alpha diversity was not significantly different between the two groups at the first three time points, but a lower alpha diversity (Shannon and Simpson indexes, *p* = 0.0176 and *p* = 0.0176, respectively) was seen in 6-month-old AD mice (Supplementary Figure S6). In addition, viral alpha diversity was not significantly different between the groups over time (Supplementary Figure S6).

**Figure 7:**
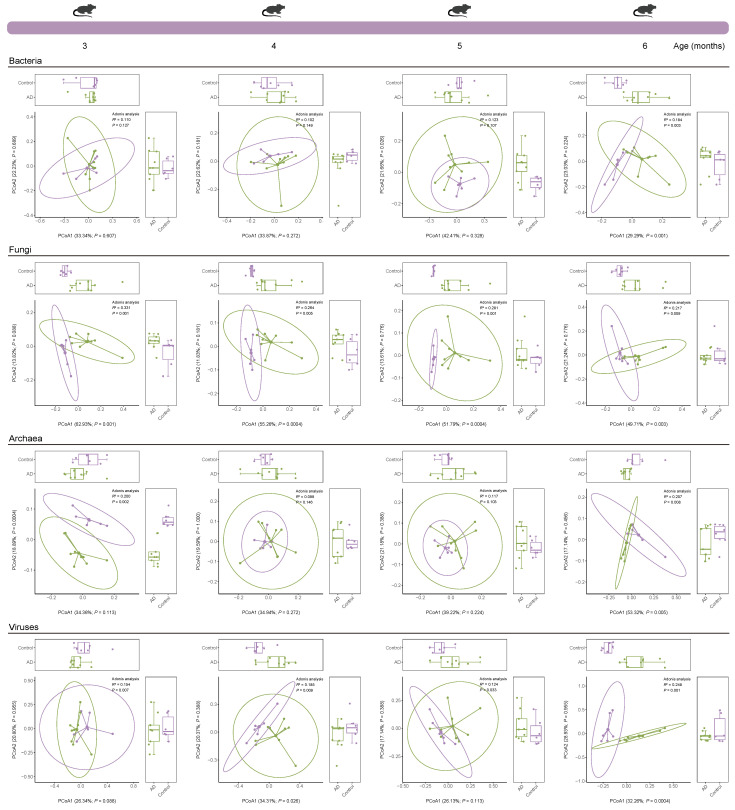
**Species-level beta diversity of mice from the Control and Alzheimer’s disease (AD) groups**. Bray–Curtis dissimilarity principal coordinate analysis (PCoA) based on bacterial, fungal, archaeal, and viral abundances of samples from the Control and AD groups. Statistical significance of differences between the two groups was determined using permutational multivariate analysis of variance (PERMANOVA, also known as adonis analysis) by 999 permutations (two-sided test). Ellipsoids in PCoA plots represent 95% confidence intervals surrounding each group. In the boxplot, the center line represents the median of the data, the lower and upper bounds of the box represent the 25th and 75th percentiles, and the lower and upper whiskers represent the minimum and maximum values.

Beta diversity based on principal coordinate analysis (PCoA) (Bray–Curtis dissimilarity) revealed a marked difference in the intestinal bacterial community structure between the two groups at 6 (*R*² = 0.184, *p* = 0.003) months of age, but not at 3, 4, and 5 months of age (Figure 7). The intestinal archaeal community structure of AD mice changed at 3 (*R*² = 0.200, *p* = 0.002) and 6 (*R*² = 0.207, *p* = 0.008) months of age, but this phenomenon was not observed in AD mice at 4 and 5 months of age (Figure 7). Remarkably, the community structure of gut fungi (*p* = 0.001, *p* = 0.005, *p* = 0.001, and *p* = 0.009, respectively) and viruses (*p* = 0.007, *p* = 0.009, *p* = 0.033, and *p* = 0.001, respectively) differed consistently between the two groups over time (Figure 7). Together, these results suggest that the development of AD is accompanied simultaneously by multi-kingdom changes in the gut microbiota composition.

### 2.5. Longitudinal and Multi-Kingdom Gut Microbiome Alterations in AD

**Figure 8:**
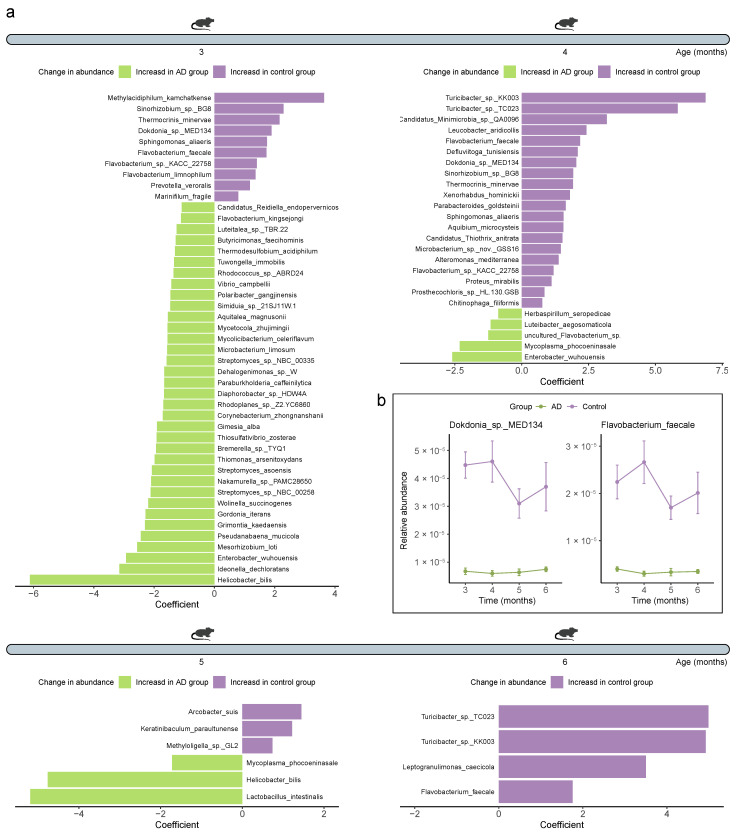
**Differentially abundant bacterial species between the Control and Alzheimer’s disease (AD) groups**. (**a**) Double bar chart showing the divergent bacterial species between the Control and AD groups, evaluated using MaAsLin2 analysis. Statistical significance was determined using a two-tailed multivariable association test, and adjusted using the false discovery rate (FDR) adjustment. Only bacterial species with *Q* < 0.001 were displayed for the first two time points, and only those with *Q* < 0.01 were shown for the last two time points. (**b**) Line chart showing the persistently different bacterial species between the Control and AD groups over time. *Q* < 0.05 was considered significant.

**Figure 9:**
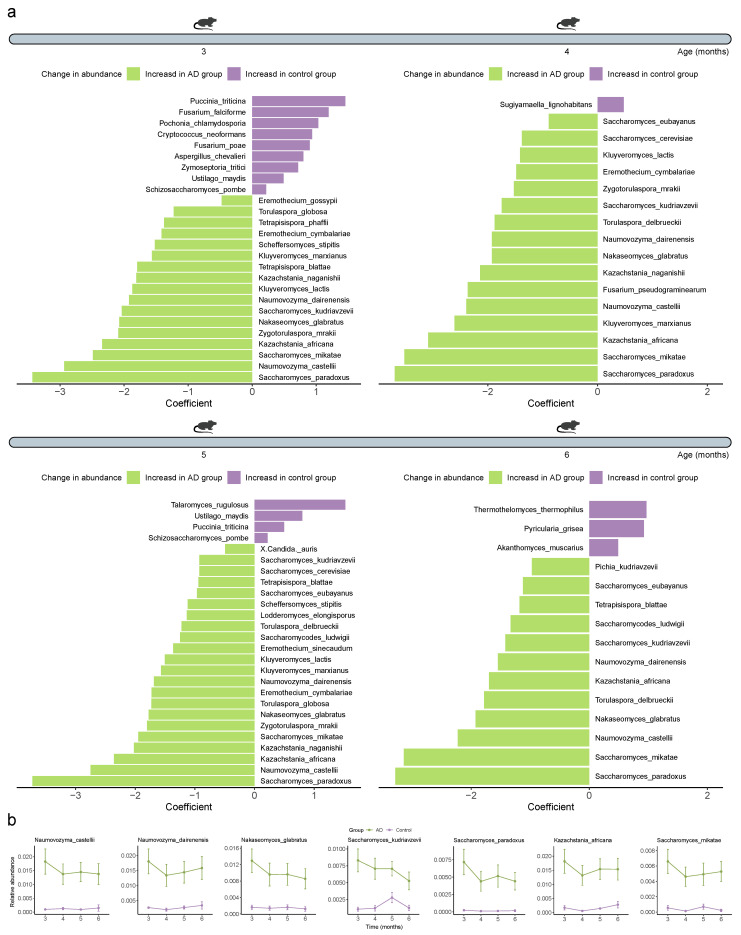
**Differentially abundant fungal species between the Control and Alzheimer’s disease (AD) groups**. (**a**) Double bar chart showing the divergent fungal species between the Control and AD groups, evaluated using MaAsLin2 analysis. Statistical significance was determined using a two-tailed multivariable association test, and adjusted using the false discovery rate (FDR) adjustment. (**b**) Line chart showing the persistently different fungal species between the Control and AD groups over time. *Q* < 0.05 was considered significant.

**Figure 10:**
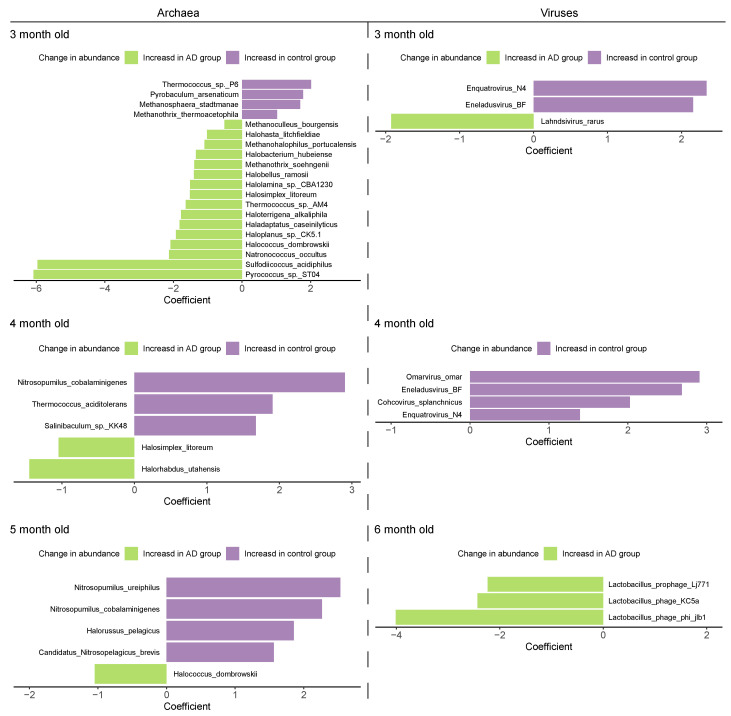
**Differentially abundant archaeal and viral species between the Control and Alzheimer’s disease (AD) groups**. Double bar chart showing the divergent archaeal and viral species between the Control and AD groups, evaluated using MaAsLin2 analysis. Statistical significance was determined using a two-tailed multivariable association test, and adjusted using the false discovery rate (FDR) adjustment. Only archaeal species with *Q* < 0.01 were displayed at the first time points. *Q* < 0.05 was considered significant.

Given that we have observed alterations in the gut microbial community structure between the Control and AD groups, especially the gut mycobiome and virome, we then tried to investigate the specific intestinal microorganisms at the species-level that are responsible for these changes. We first determined the relative abundance of which intestinal bacteria was differentially abundant between the healthy and diseased mice. We observed 843 (8.17%), 214 (2.08%), 112 (1.08%), and 8 (0.08%) differential bacterial taxa between the two groups when the mice were 3, 4, 5, and 6 months old, respectively (*Q* < 0.05) (Supplementary Table S1). The intestinal bacteria of 3-, 4-, 5-, and 6-month-old AD mice contained significantly fewer *Methylacidiphilum kamchatkense*, *Turicibacter* sp. KK003, *Arcobacter suis*, and *Turicibacter* sp. TC023, respectively (*Q* < 0.01) (Figure 8a). We further identified two bacterial species (*Dokdonia* sp. MED134 and *Flavobacterium faecale*) that consistently differed across all time points, with their abundances being continuously lower in the AD mice than in the Control mice (*Q* < 0.05) (Figure 8b). In addition to the 26 (25.24%), 17 (16.35%), 26 (25.00%), and 15 (14.56%) significantly different fungal species found at different time points, respectively (*Q* < 0.05) (Figure 9a, Supplementary Table S2), we also detected seven consistently significantly distinct species across all time points, including *Naumovozyma castellii*, *Naumovozyma dairenensis*, *Nakaseomyces glabratus*, *Saccharomyces kudriavzevii*, *Saccharomyces paradoxus*, *Kazachstania africana*, and *Saccharomyces mikatae*, all of which were notably elevated in AD mice (*Q* < 0.05) (Figure 9b). For archaea, 49 (10.65%), 5 (1.09%), 5 (1.08%), and 0 (0%) differential species were identified at different time points, respectively (*Q* < 0.05) (Figure 10, Supplementary Table S3). Specifically, *Pyrococcus* sp. ST04, *Halorhabdus utahensis*, and *Halococcus dombrowskii* prevailed in the 3-, 4-, and 5-month-old AD mice, respectively, whereas *Thermococcus* sp. P6, *Nitrosopumilus cobalaminigenes*, and *Nitrosopumilus ureiphilus* dominated in healthy controls matched for age, respectively (*Q* < 0.05) (Figure 10). In addition, three (0.60%), four (0.82%), zero (0%), and three (0.65%) differential viral species were identified in the mice at 3, 4, 5, and 6 months of age, respectively, among which *Lactobacillus prophage Lj771*, *Lactobacillus phage KC5a*, and *Lactobacillus phage phi jlb1* were prevalent in 6-month-old AD mice (*Q* < 0.05) (Figure 10, Supplementary Table S4). Collectively, these data suggest that the gut bacteriome, mycobiome, archaeome, and virome are altered during the development and progression of AD.

### 2.6. Slight Fluctuations in Microbial Function in AD

Since we observed shifts in four-kingdom microbiome composition, we then asked whether the functional potential of the gut microbiome changed. To address this, we investigated the functional alternations in MetaCyc pathways, Kyoto Encyclopedia of Genes and Genomes (KEGG) Orthology (KO) genes, Gene Ontology (GO) terms, and evolutionary genealogy of genes: Non-supervised Orthologous Groups (eggNOG) genes based on their relative abundance data. Almost no significant differences in functional alpha diversity, represented by Shannon and Simpson indexes, were observed between the Control and AD groups at each time point (Supplementary Figure S7). Similar results were obtained in terms of functional beta diversity based on non-metric multidimensional scaling (NMDS) analysis using Bray–Curtis dissimilarity (Supplementary Figure S8). Despite the fact that we did not observe overall functional changes in the gut microbiome, we still sought to identify potential functional differences between the Control and AD groups. MaAsLin2 analysis returned 27 (8.1%, *Q* < 0.25), 9 (2.5%, *Q* < 0.5), 16 (4.7%, *Q* < 0.25), and 17 (5.2%*, Q* < 0.25) differential MetaCyc pathways between the two groups at months 3, 4, 5, and 6, respectively (Supplementary Figure S9). In addition, differential KO genes (10 (0.3%, *Q* < 0.05), 8 (0.3%, *Q* < 0.25), 8 (0.3%, *Q* < 0.05), and 21 (0.8%, *Q* < 0.2)), GO terms (18 (0.4%, *Q* < 0.05), 15 (0.4%, *Q* < 0.25), 29 (0.7%, *Q* < 0.5), and 19 (0.5%, *Q* < 0.2)), and eggNOG genes (32 (0.2%, *Q* < 0.01), 28 (0.2%, *Q* < 0.05), 10 (0.1%, *Q* < 0.05), and 12 (0.1%, *Q* < 0.25)) between the Control and AD groups at every time point were also determined (Supplementary Figures S10–S12). In other words, our results suggest that the functional potential of the gut microbiome is partially significantly altered in the development of AD.

## 3. Discussion

In this paper, we longitudinally deciphered the blueprint of the gut microbiome of fecal samples from healthy and AD mice, including bacteriome, mycobiome, archaeome, and virome. Our results showed that not only the gut bacteriome, but also the gut mycobiome, archaeome, and virome were altered during the development and progression of AD. Insights into the longitudinal and multi-kingdom dynamics of the gut microbiome will be critical for guiding rational microbiome-based therapies targeting AD.

Research interest in the role of the gut microbiome in human health and disease has been increasingly appreciated, including in relation to AD [15]. While disturbances in the gut microbiome (the gut bacteriome) have been observed in both animals and humans with AD [3], longitudinal and multi-kingdom gut microbiome profiling prior to the onset of disease pathology are still lacking. Given the variations in the timing of the clinical diagnosis of AD patients (even if a patient is clinically diagnosed with AD, it can be difficult to determine the patient’s initial pathological starting point) and the susceptibility of the human gut microbiota to environmental factors such as diet and lifestyle [16], APP/PS1 transgenic mice, one of the most widely used AD animal models [17], were selected as the subjects of this study. Consistent with previous studies [18], we also observed premature death in AD mice, which could be attributed to seizures [12]. The 90-day survival rate of APP/PS1 mice was 90%, which provided a reference for determining the number of experimental animals for future studies. Remarkably, there was no significant difference in body weight between the Control and AD groups at the age of 6 months. The appearance of Aβ plaques in the hippocampus of APP/PS1 mice around 6 months of age has become widely accepted [19], which was also observed in our current study. Collectively, the APP/PS1 mouse is a suitable animal model of AD for the elaboration of current scientific questions.

We identified 41,222 bacterial, 414 fungal, 1836 archaeal, and 1916 viral species from AD mice and healthy mice across all time points using shotgun metagenomic sequencing, providing multi-kingdom species information related to AD. Microorganisms residing in the gut not only affect the host alone [20,21], but also interact with other members of the gut microbiota to affect the host, prompting us to explore the interactions between microbiota in the gut. We observed more complex bacteria–bacteria (except at 3 months of age) and fungi–fungi interactions, as well as simpler archaea–archaea (except at 5 months of age) and virus–virus (except at 4 months of age) interactions in AD mice. A previous study explores changes in the dynamic gut bacterial network in APP/PS1 mice and healthy controls and finds that the network in AD mice is more complex, which is largely similar to our findings [22]. Currently, investigation into the state of the gut microbiome network in AD is still in its infancy, particularly in patients with AD. Moreover, causality between the gut microbiome network and AD is lacking and needs to be investigated in future studies.

A recent review article summarizes the findings from 11 observational studies investigating alterations in the gut microbiota and finds that most studies (63.6%, 7/11) show little difference in the alpha diversity of the gut microbiota between healthy and AD mice, whereas the results for beta diversity indicate the opposite trend (72.7%, 8/11) [3]. In line with this, our results also showed that the gut bacterial diversity between AD and healthy mice was not always significantly different at various time points. The genera *Ruminococcus* (decreased in AD mice from four studies), *Desulfovibrio* and *Lactobacillus* (increased in AD mice from three studies), *Allobaculum* (decreased and increased in AD mice from three studies and two studies, respectively), and *Akkermansia* (decreased and increased in AD mice from two studies and one study, respectively) are the most frequently reported differentially abundant gut taxa between the healthy and AD mice in these 11 selected studies [3]. Considering the inability of 16S rRNA gene sequencing, which is widely used in these observational studies to identify microbial taxa at a finer level, higher-resolution shotgun metagenomic sequencing was implemented in our study, which would provide more information on species-level changes. Shotgun metagenomic sequencing showed more *Helicobacter bilis* and *Ideonella dechloratans* but fewer *Methylacidiphilum kamchatkense* and *Sinorhizobium* sp. BG8 in 3-month-old AD mice; more *Enterobacter wuhouensis* and *Mycoplasma phocoeninasale* but fewer *Turicibacter* sp. KK003 and *Turicibacter* sp. TC023 in 4-month-old AD mice; more *Lactobacillus intestinalis* and *Helicobacter bilis* but fewer *Arcobacter suis* and *Keratinibaculum paraultunense* in 5-month-old AD mice; and fewer *Turicibacter* sp. TC023 and *Turicibacter* sp. KK003 in 6-month-old AD mice. Consistent with our findings, a previous study shows that 18-month-old 5 × FAD mice, another AD mouse model, contain significantly less *Turicibacter* species [23]. However, the role of other differential bacteria in AD development needs to be further investigated.

Few studies exist that explore the changes in nonbacterial members in AD. Our study further investigated the gut fungal, archaeal, and viral communities via high-throughput sequencing. We identified 84 differential fungal species between the two groups, of which 7 species appeared at each time point, including higher levels of *Naumovozyma castellii*, *Naumovozyma dairenensis*, *Nakaseomyces glabratus*, *Saccharomyces kudriavzevii*, *Saccharomyces paradoxus*, *Kazachstania africana*, and *Saccharomyces mikatae* in AD mice. The gut archaeal microbiota of AD mice was characterized by higher *Pyrococcus* sp. ST04 and *Sulfodiicoccus acidiphilus*, but lower *Thermococcus* sp. P6 and *Pyrobaculum arsenaticum* (3 months old); higher *Halorhabdus utahensis* and *Halosimplex litoreum* but lower *Nitrosopumilus cobalaminigenes* and *Thermococcus aciditolerans* (4 months old); and higher *Halococcus dombrowskii*, but lower *Nitrosopumilus ureiphilus* and *Nitrosopumilus cobalaminigenes* (5 months old). In addition, 10 differential viral species were identified between the Control and AD groups. Specifically, the gut viral microbiota of AD mice had more *Lahndsivirus rarus* (3 months old), as well as more *Lactobacillus prophage Lj771*, *Lactobacillus phage KC5a*, and *Lactobacillus phage phi jlb1* (6 months old). An underrepresentation of *Enquatrovirus N4* and *Eneladusvirus BF* (3 and 4 months old) was also observed in AD mice. Indeed, two previous independent studies have described changes in the gut fungal communities in a genetically modified mouse model of AD and patients with mild cognitive impairment (the prodromal phase of AD) by biomarker sequencing (internal transcribed spacer region) [24,25]. The former finds that the family *Dipodascaceae* is significantly increased in AD mice [24], while the latter finds that the genera *Botrytis*, *Kazachstania*, *Phaeoacremonium*, and *Cladosporium* are significantly increased in patients with mild cognitive impairment [25]. Our study further identified significant increases in *Kazachstania naganishii* and *Kazachstania africana* belonging to the *Kazakhstania* genus in AD mice at the species level. No studies have yet described the changes in gut viral and archaeal communities in the context of AD, and the differential archaeal and viral species we identified in the current study inform future related research.

Although we identified longitudinal and multi-kingdom changes in the gut microbiome, we found that there were few shared differential species across all time points. The age-related variations in the differential intestinal microbiota identified between the Control and AD groups underscore the importance of a longitudinal design in unraveling the evolution of gut microbiota-related changes in AD. Hence, it can be further speculated that evidence for the administration of a key single microorganism or metabolite selected through multi-omics approaches at a single sampling point to experimental animals to validate its ameliorative or deteriorating effect on disease may be undermined by the highly dynamic nature of the gut microbiome over time, posing a substantial challenge to elucidating the role of the gut microbiome in disease at the per-microbe scale. Although our study concluded that longitudinal and multi-kingdom gut microbiome alterations were present in the setting of AD, it remains to be studied whether such changes are a cause, a consequence, or are incidental to the disease. In addition, two recent review articles summarizing fungal infection in the brain of patients with AD have emphasized the important role of extraintestinal fungi in AD [26,27]. Whether gut microbiota can migrate from the gut to remote extraintestinal sites such as the brain tissue to exert influences on the host is worth investigating.

We compared the functional potential of the gut microbiome between the Control and AD groups, including MetaCyc pathways, KO genes, GO terms, and eggNOG genes. Although we did not observe overall changes in the functional alternations, numerous differential functional pathways and genes were identified between the two groups at different time points. These results underscored the fact that the functional potential of the gut microbiome was also partially altered.

Our study has some limitations. First, despite male mice being the most widely used in the realm of AD research, we did not characterize the gut microbiome configuration of female mice, which warrants further investigation. Second, while neuropathological features and cognitive function are two important aspects for evaluating APP/PS1 mice (especially the former), we did not perform behavioral tests on these mice, mainly due to the ongoing COVID-19 pandemic and stringent containment policies for this disease in China during the animal experiment. Given the fact that the manifestation of cognitive impairment lags decades behind the neuropathological changes in AD patients [28], further research is still needed to determine the timing of behavioral changes in AD animal models. Third, while sustained gut microbiota dysbiosis was observed prior to the pathological manifestation of AD mice in our study, whether or not the gut microbiota is a major driver of AD still requires further investigation. Fourth, the current study is based on an AD animal model, and the gut microbiome characteristics obtained from this model are unlikely to generalize to those of AD patients. Well-designed, large-scale observational studies are needed to investigate longitudinal and multi-kingdom gut microbiome profiling in AD patients.

In conclusion, we report for the first time the longitudinal and multi-kingdom gut microbiome alterations in a mouse model of AD using higher-resolution shotgun metagenomic sequencing. Our study highlights the close relationship between the multi-kingdom gut microbiome and the onset and progression of AD, and this correlation, as well as potential causality, needs to be further investigated in future studies.

## 4. Materials and Methods

### 4.1. Animals

The animal experiment was approved by the Laboratory Animal Welfare and Ethics Committee of Inner Mongolia Agricultural University (No. NND2023077), following the ARRIVE guidelines 2.0 [29]. Ten 11-week-old specific pathogen-free (SPF) male APP/PS1 transgenic mice of a C57BL/6J background, as well as six age- and sex-matched SPF wild-type C57BL/6J mice, were purchased from Beijing Huafukang Bioscience Co., Ltd. (Beijing, China). Mice were housed under individually ventilated cages where they had free access to sterile water and food, with a temperature of 22  ±  2  °C, a relative humidity of 45  ±  10%, and a dark–light cycle of 12 h. After a 7-day acclimatization period, mice were divided into two groups: (1) Control group (n = 6): wild-type mice; (2) AD group (n = 10): APP/PS1 mice. No mice were subjected to any interventions. APP/PS1 mice express a chimeric mouse/human amyloid precursor protein (Mo/HuAPP695swe) and a mutant human presenilin 1 (PS1-dE9) [30], developing Aβ deposits in the brain around 6 months of age [19], which can mimic the pathology of AD and are widely used in AD research.

### 4.2. Sample Collection

Fecal samples were collected from mice at the age of 3, 4, 5, and 6 months and stored at −80 °C immediately after sampling. The body weight of the mice was simultaneously recorded. In addition, the number of dead mice was recorded daily. The brain tissue was collected from mice at the age 6 months.

### 4.3. Immunohistochemistry

The removed brain was fixed in 4% paraformaldehyde for 24 h, dehydrated with graded alcohol, and coronally cut into 4 μm sections on a rotary microtome (HistoCore BIOCUT, Leica Biosystems, Wetzlar, Germany). The antibody used was anti-beta amyloid 1–42 antibody (ab201060, 1:500 dilution, Abcam, Cambridge, UK). Slides were scanned using Pannoramic 250 FLASH III (3DHISTECH, Budapest, Hungary) and representative images were taken using ImageJ (version 1.54f).

### 4.4. DNA Extraction, Shotgun Metagenomic Sequencing, and Bioinformatics

DNA from fecal samples was extracted for metagenomic sequencing using a QIAamp Fast DNA Stool Mini Kit (Qiagen, Hilden, Germany). The quality of isolated DNA was evaluated using a NanoDrop One Spectrophotometer (Thermo Fisher Scientific, Waltham, MA, USA) and the qualified DNA was stored at −80 °C. Libraries were prepared using a NEBNext Ultra DNA Library Prep Kit for Illumina (New England Biolabs, Ipswich, MA, USA). The constructed library quality was assessed using a Qubit 2.0 Fluorometer (Thermo Fisher Scientific, Waltham, MA, USA). The qualified libraries were pooled and sequenced on a NovaSeq 6000 System (Illumina, San Diego, CA, USA) with a PE150 strategy. Raw data were subjected to filtering of low-quality and host-contaminating reads using Trimmomatic [31] (version 0.39) and Bowtie2 [32] (version 2.5.1), respectively, within the KneadData tool (version 0.10.0; reference database: mouse_C57BL). The remaining reads were taxonomically classified using Kraken2 [33] (version 2.1.3; reference database: bacterial, fungal, archaeal, and viral databases constructed using the kraken2-build command), followed by the estimation of the abundance of each taxon using Bracken [34] (version 2.9; reference database: bacterial, fungal, archaeal, and viral k-mer distributions constructed using the bracken-build command). In addition, the high-quality reads were functionally annotated using HUMAnN3 [35] (version 3.9, ChocoPhlAn database: full chocophlan.v201901_v31; UniRef database: uniref50_201901b_full; Utility database: full_mapping_v201901b). Gene families generated by HUMAnN3 were regrouped into other functional categories, including Kyoto Encyclopedia of Genes and Genomes (KEGG) Orthology (KO), Gene Ontology (GO), and evolutionary genealogy of genes: Non-supervised Orthologous Groups (eggNOG).

### 4.5. Statistical Analysis

All statistical analysis and visualization were achieved using R [36] (version 4.4.0), and the graphics were combined with Adobe Illustrator [37] (version 28.0).

General data. The overall survival of the two groups was compared using a log-rank test using survival package [38] (version 3.7-0) and the results were visualized using the survminer package [39] (version 0.4.9). Data on body weight change and histopathology score were tested for independence, normality, and homogeneity of variance using stats package [36] (version 4.4.0), and were then statistically analyzed using either two-sided Student’s *t*-test (parametric test) or two-sided Wilcoxon rank-sum test (nonparametric test), depending on the tested results. Finally, the data on body weight change and histopathology score were visualized using the ggpubr package [40] (version 0.6.0) and ggbeeswarm package [41] (version 0.7.2), respectively. Data are represented as mean  ±  standard error of the mean (s.e.m). Significance levels are as follows: * *p*  <  0.05; ** *p*  <  0.01; *** *p*  <  0.001; **** *p*  <  0.0001; and NS (not significant).

Metagenomic data. Taxonomic and functional alpha diversity metrics between the two groups, represented by Shannon and Simpson indexes, were calculated using the vegan package [42] (version 2.6-8), and were then statistically analyzed using two-sided Wilcoxon rank-sum test, and finally visualized using the ggpubr package [40] (version 0.6.0). Taxonomic and functional beta diversity metrics between the two groups were calculated with the vegan package [42] (version 2.6-8) in combination with the ape package [43] (version 5.8), and were then statistically analyzed via the permutational multivariate analysis of variance (PERMANOVA, also known as adonis analysis, Bray–Curtis dissimilarity, permutations = 999) using the vegan package [42] (version 2.6-8), and were finally visualized using the ggpubr package [40] (version 0.6.0) and ggplot2 package [44] (version 3.5.1), respectively. The composition of gut microbiota at the species level was visualized using the ggplot2 package [44] (version 3.5.1) and ggtree package [45] (version 3.12.0). The differential species and functional potential between the two groups were determined by MaAsLin2 analysis using the Maaslin2 package [46] (version 1.18.0), with the former visualized using the ggplot2 package [44] (version 3.5.0) and the latter visualized using the pheatmap package [47] (version 1.0.12). Species-level ecological network analysis was performed using the ggClusterNet package [48] based on Spearman’s correlation (version 0.1.0; *R* threshold = 0.8, *p* threshold = 0.05, method = spearman). Only species with a mean relative abundance of at least 0.01% were used for network construction. The obtained edge and node data were further visualized via the ggplot2 package [44] (version 3.5.0). Note that the *p* value here is the false discovery rate (FDR)-corrected value, namely the *Q* value.

## Data Availability

Raw sequencing reads are available via the NCBI Sequence Read Archive with the accession number PRJNA1116578.

## Supplementary Materials

The following supporting information can be downloaded at: https://www.mdpi.com/article/10.3390/ijms252111472/s1.
