# Supplementary material for: Longitudinal and Multi-Kingdom Gut Microbiome Alterations in a Mouse Model of Alzheimer’s Disease
Source: Int J Mol Sci. 2024 Oct 25;25(21):11472. doi: 10.3390/ijms252111472 (PMC11546883; doi:10.3390/ijms252111472)
Supplement: Supplementary file 1 [file ijms-25-11472-s001.zip › Captions of Figures and Tables.pdf]

Supplementary Figure S1. Phylogenetic tree showing the selected gut bacterial species of mice from the Control and Alzheimer's disease (AD) groups. a, 3-month-old. **b**, 4-month-old. **c**, 5-month-old. **d**, 6-month-old. The color blocks on the taxonomic tree represent phyla. The circles on the branches of the taxonomic tree from outside to inside are genus, family, order, class, phylum, and kingdom. The outermost layer of the graph represents the bacterial species identified across all samples. The squares represent the distribution of the relative abundance of each identified bacterial species in each sample, with their size proportional to their relative abundance. Only top 100 bacterial species ranked by mean relative abundance across all samples were shown.

Supplementary Figure S2. Phylogenetic tree showing the selected gut fungal species of mice from the Control and Alzheimer's disease (AD) groups. a, 3-month-old. **b**, 4-month-old. **c**, 5-month-old. **d**, 6-month-old. The color blocks on the taxonomic tree represent phyla. The circles on the branches of the taxonomic tree from outside to inside are genus, family, order, class, phylum, and kingdom. The outermost layer of the graph represents the fungal species identified across all samples. The squares represent the distribution of the relative abundance of each identified fungal species in each sample, with their size proportional to their relative abundance. Only top 100 fungal species ranked by mean relative abundance across all samples were shown.

Supplementary Figure S3. Phylogenetic tree showing the selected gut archaeal species of mice from the Control and Alzheimer's disease (AD) groups. a, 3-month-old. **b**, 4-month-old. **c**, 5-month-old. **d**, 6-month-old. The color blocks on the taxonomic tree represent phyla. The circles on the branches of the taxonomic tree from outside to inside are genus, family, order, class, phylum, and kingdom. The outermost layer of the graph represents the archaeal species identified across all samples. The squares represent the distribution of the relative abundance of each identified archaeal species in each sample, with their size proportional to their relative abundance. Only top 100 archaeal species ranked by mean relative abundance across all samples were shown.

Supplementary Figure S4. Phylogenetic tree showing the selected gut viral species

of mice from the Control and Alzheimer's disease (AD) groups. a, 3-month-old. **b**, 4-month-old. **c**, 5-month-old. **d**, 6-month-old. The color blocks on the taxonomic tree represent phyla. The circles on the branches of the taxonomic tree from outside to inside are genus, family, order, class, phylum, and kingdom. The outermost layer of the graph represents the viral species identified across all samples. The square represents the distribution of the relative abundance of each identified viral species in each sample, with their size proportional to their relative abundance. Only top 100 viral species ranked by mean relative abundance across all samples were shown.

Supplementary Figure S5. Overview of the dominant multi-kingdom species composition in per mouse. Distribution of the most abundant bacterial, fungal, archaeal, and viral species in each mouse from the Control and Alzheimer's disease (AD) groups at months 3, 4, 5, and 6 based on mean relative abundance. The species ranked from 11th to the last based on mean relative abundance across all samples were grouped as ‘Others’.

Supplementary Figure S6. Species-level alpha diversity of mice from the Control and Alzheimer's disease (AD) groups. Bacterial, fungal, archaeal, and viral alpha diversity measured by Shannon and Simpson indexes of mice from the Control and AD groups at different time points. Data are represented as mean ± standard deviation (s.d.). $*P < 0.05$, $**P < 0.01$, $***P < 0.001$; NS, not significant. The box denotes the 25-75th percentiles and the central mark indicates the median. Statistical significance of differences between the two groups was determined by two-sided Wilcoxon rank-sum test.

Supplementary Figure S7. Functional alpha diversity of mice from the Control and Alzheimer's disease (AD) groups. The alpha diversity of MetaCyc pathways, Kyoto Encyclopedia of Genes and Genomes (KEGG) Orthology (KO) genes, Gene Ontology (GO) terms, and evolutionary genealogy of genes: Non-supervised Orthologous Groups (eggNOG) genes measured by Shannon and Simpson indexes between the Control and AD groups at the age of 3, 4, 5, and 6 months. Data are

represented as mean ± standard deviation (s.d.). *$P < 0.05$; NS, not significant. The box denotes the 25-75th percentiles and the central mark indicates the median. Statistical significance of differences between the two groups was determined by two-sided Wilcoxon rank-sum test.

Supplementary Figure S8. Functional beta diversity of mice from the Control and Alzheimer's disease (AD) groups. Non-metric multidimensional scaling (NMDS) analysis of MetaCyc pathways, Kyoto Encyclopedia of Genes and Genomes (KEGG) Orthology (KO) genes, Gene Ontology (GO) terms, and evolutionary genealogy of genes: Non-supervised Orthologous Groups (eggNOG) genes based on Bray-Curtis dissimilarity between the Control and AD groups at the age of 3, 4, 5, and 6 months. Statistical significance of differences between the two groups was determined using permutational multivariate analysis of variance (PERMANOVA, also known as adonis analysis) by 999 permutations. Ellipsoids in NMDS plots represent 95% confidence intervals surrounding each group.

Supplementary Figure S9. Differentially abundant MetaCyc pathways between the Control and Alzheimer's disease (AD) groups. The differential MetaCyc pathways between the Control and AD groups were identified via MaAsLin2 analysis at the age of 3 ($Q < 0.25$), 4 ($Q < 0.5$), 5 ($Q < 0.25$), and 6 ($Q < 0.25$) months. Statistical significance was determined by two-tailed multivariable association test, and adjusted by the false discovery rate (FDR) adjustment. A different Q threshold was considered statistically significant. Red squares represent the relative abundance of MetaCyc pathways in the sample was significantly increased, whereas blue squares represent the relative abundance of MetaCyc pathways in the sample was significantly decreased.

Supplementary Figure S10. Differentially abundant Kyoto Encyclopedia of Genes and Genomes (KEGG) Orthology (KO) genes between the Control and Alzheimer's disease (AD) groups. The differential KO genes between the Control and AD groups were identified via MaAsLin2 analysis at the age of 3 ($Q < 0.05$), 4 ($Q < 0.25$), 5 ($Q < 0.05$), and 6 ($Q < 0.2$) months. Statistical significance was determined by

two-tailed multivariable association test, and adjusted by the false discovery rate (FDR) adjustment. A different *Q* threshold was considered statistically significant. Red squares represent the relative abundance of KO genes in the sample was significantly up-regulated, whereas blue squares represent the relative abundance of KO genes in the sample was significantly down-regulated.

Supplementary Figure S11. Differentially abundant Gene Ontology (GO) terms between the Control and Alzheimer's disease (AD) groups. The differential GO terms between the Control and AD groups were identified via MaAsLin2 analysis at the age of 3 ($Q < 0.05$), 4 ($Q < 0.25$), 5 ($Q < 0.5$), and 6 ($Q < 0.2$) months. Statistical significance was determined by two-tailed multivariable association test, and adjusted by the false discovery rate (FDR) adjustment. A different *Q* threshold was considered statistically significant. Red squares represent the relative abundance of GO terms in the sample was significantly up-regulated, whereas blue squares represent the relative abundance of GO terms in the sample was significantly down-regulated.

Supplementary Figure S12. Differentially abundant evolutionary genealogy of genes: Non-supervised Orthologous Groups (eggNOG) genes between the Control and Alzheimer's disease (AD) groups. The differential eggNOG genes between the Control and AD groups were identified via MaAsLin2 analysis at the age of 3 ($Q < 0.01$), 4 ($Q < 0.05$), 5 ($Q < 0.05$), and 6 ($Q < 0.25$) months. Statistical significance was determined by two-tailed multivariable association test, and adjusted by the false discovery rate (FDR) adjustment. A different *Q* threshold was considered statistically significant. Red squares represent the relative abundance of eggNOG genes in the sample was significantly up-regulated, whereas blue squares represent the relative abundance of eggNOG genes in the sample was significantly down-regulated.

Supplementary Table S1. List of differentially abundant bacterial species between the Control group and Alzheimer's disease (AD) groups

Supplementary Table S2. List of differentially abundant fungal species between the Control group and Alzheimer's disease (AD) groups

Supplementary Table S3. List of differentially abundant archaeal species between the Control group and Alzheimer's disease (AD) groups

Supplementary Table S4. List of differentially abundant viral species between the Control group and Alzheimer's disease (AD) groups
