# Supplementary figures and images for: Longitudinal and Multi-Kingdom Gut Microbiome Alterations in a Mouse Model of Alzheimer’s Disease
Source: Int J Mol Sci. 2024 Oct 25;25(21):11472. doi: 10.3390/ijms252111472 (PMC11546883; doi:10.3390/ijms252111472)

a

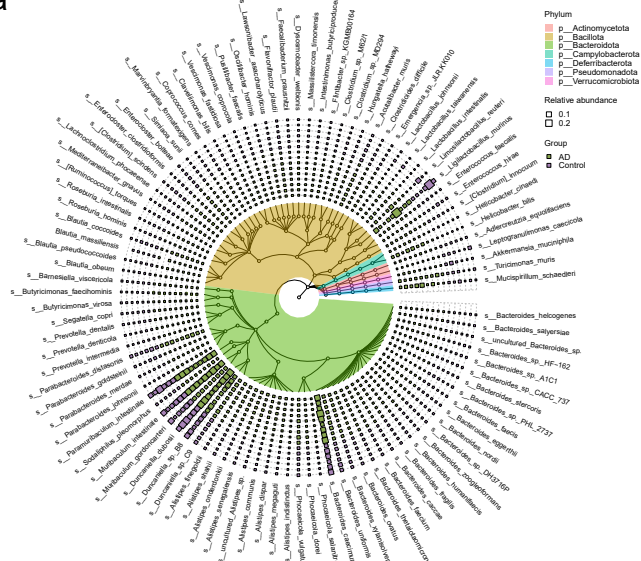

b

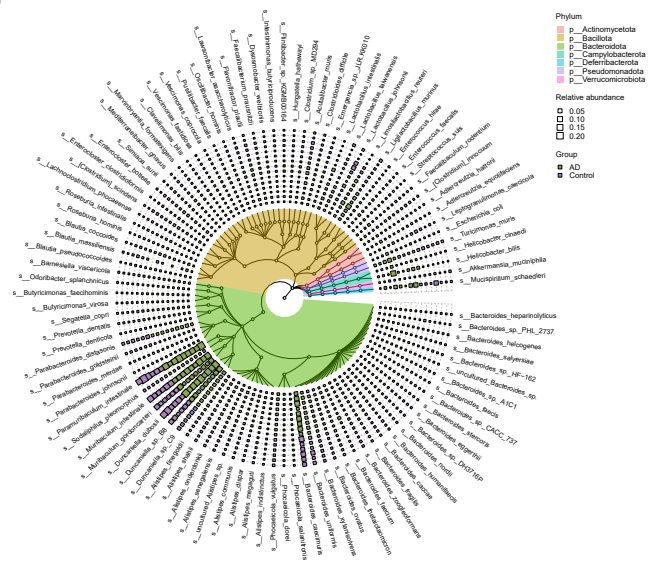

c

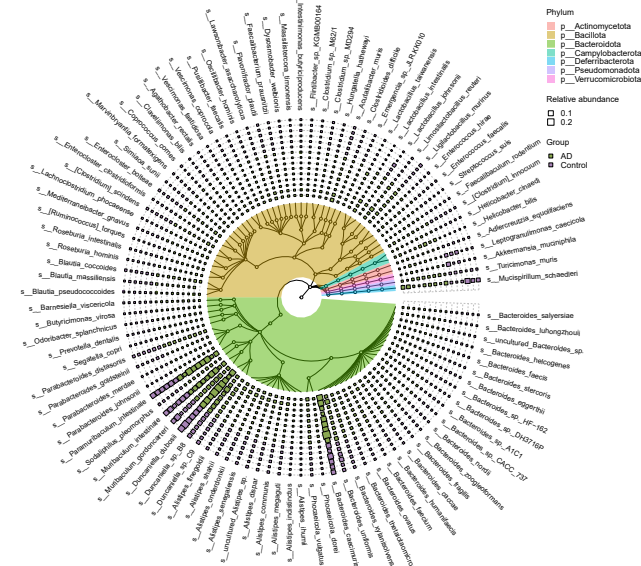

d

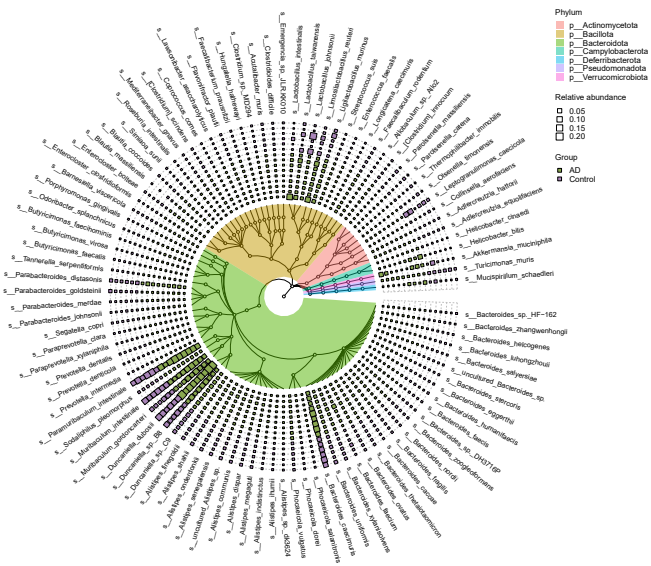

Supplement: Supplementary file 1 [file ijms-25-11472-s001.zip › Supplementary Figure S1.pdf]

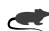

3

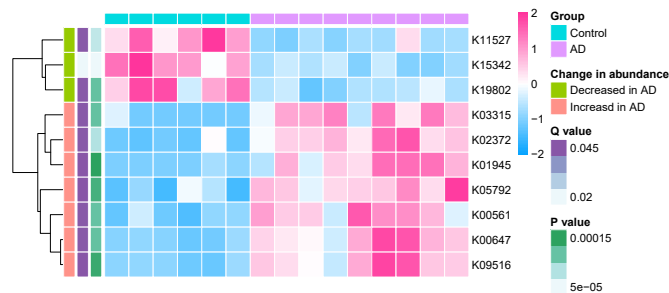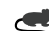

4

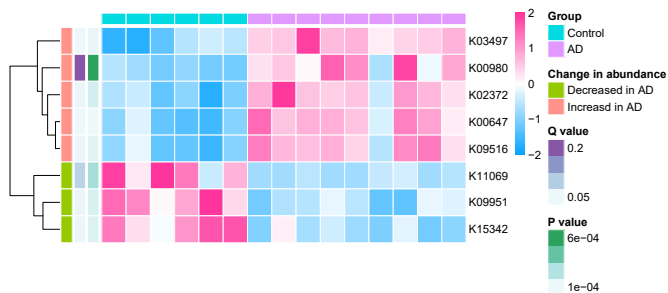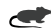

5

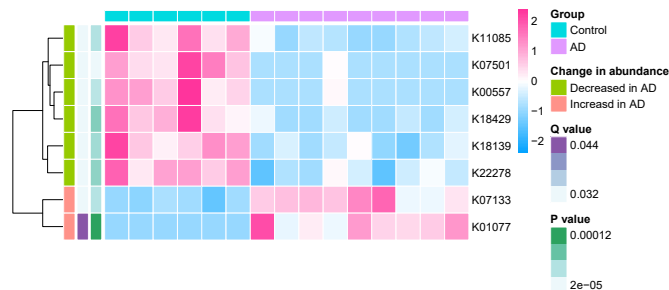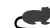

6

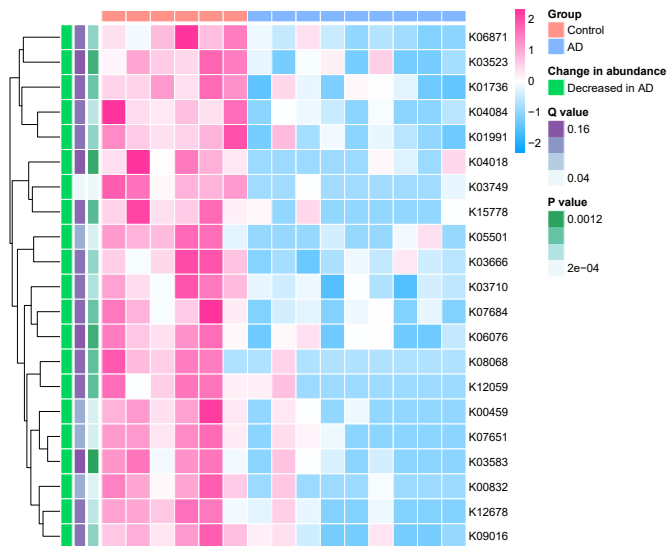

Supplement: Supplementary file 1 [file ijms-25-11472-s001.zip › Supplementary Figure S10.pdf]

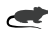

3

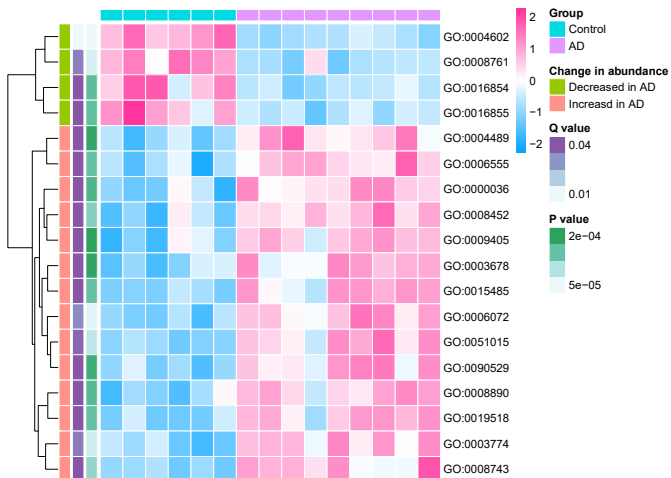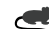

4

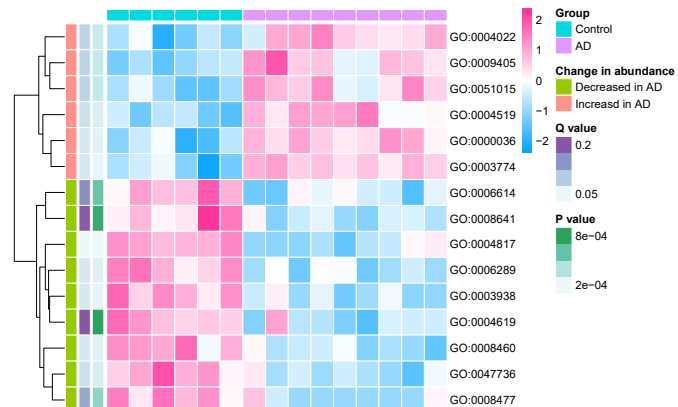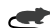

5

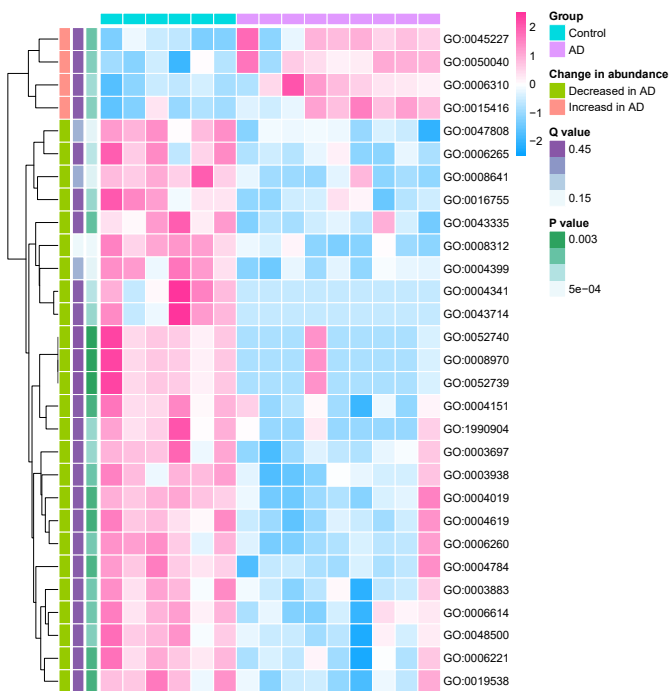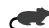

6

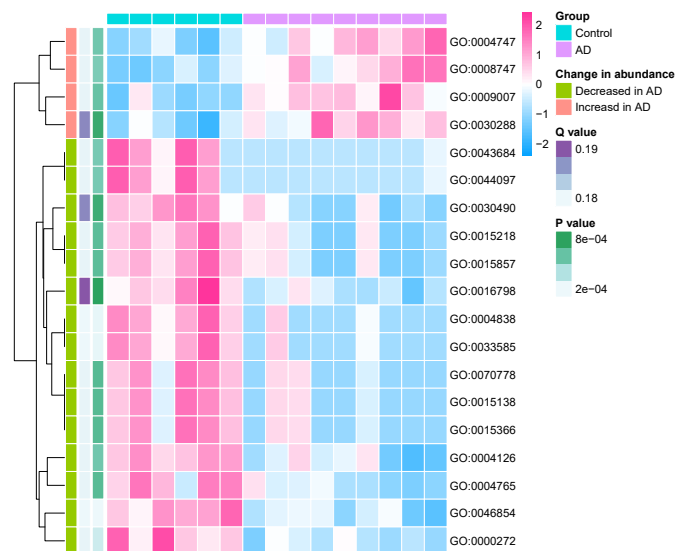

Supplement: Supplementary file 1 [file ijms-25-11472-s001.zip › Supplementary Figure S11.pdf]

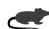

3

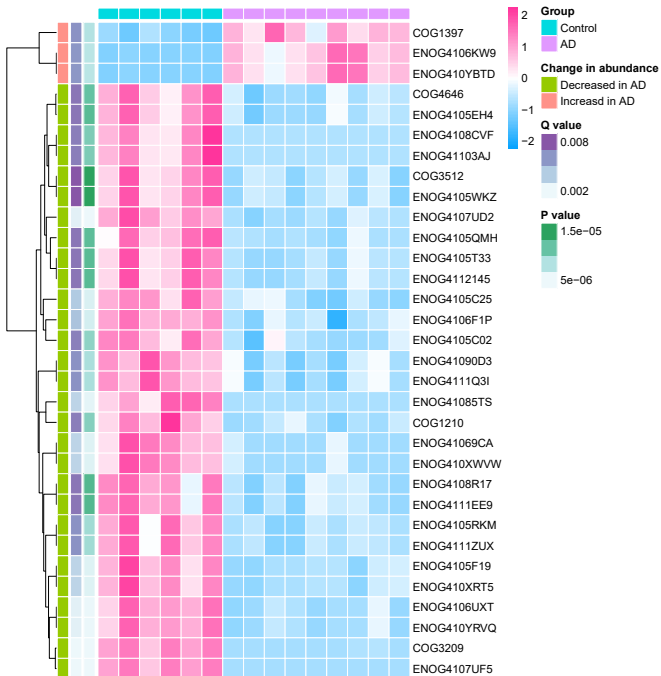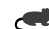

4

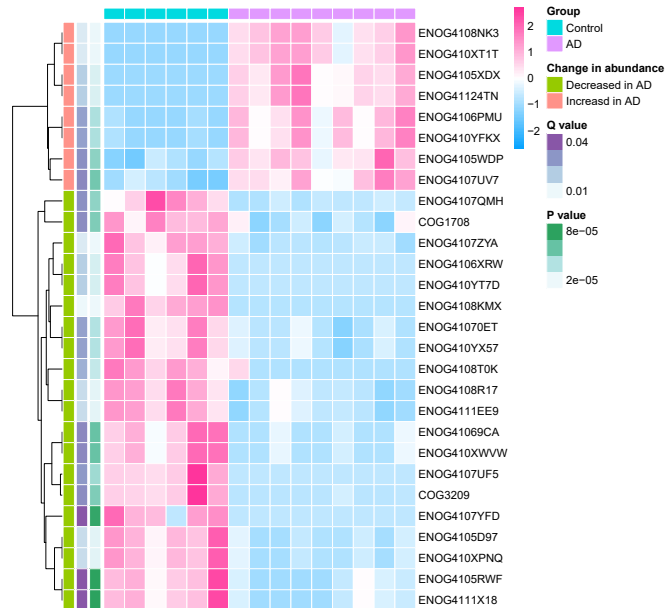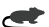

5

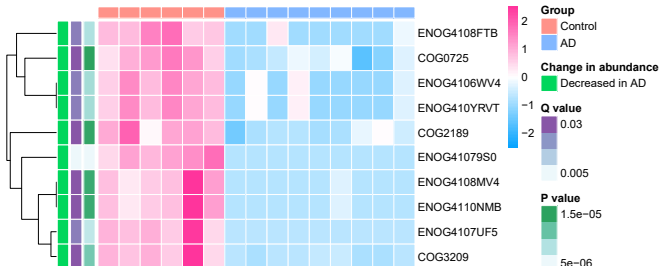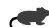

6

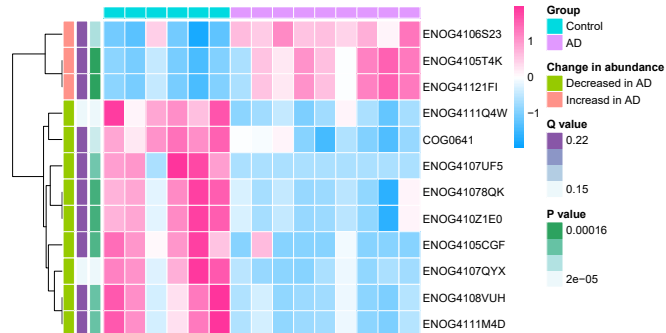

Supplement: Supplementary file 1 [file ijms-25-11472-s001.zip › Supplementary Figure S12.pdf]

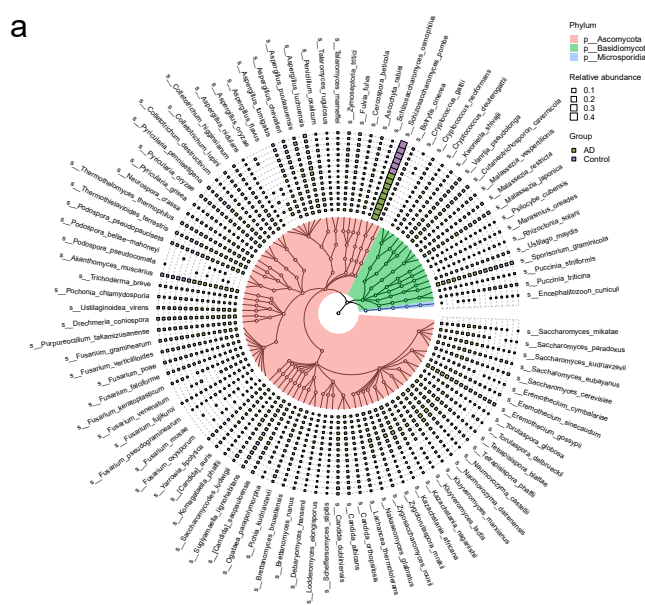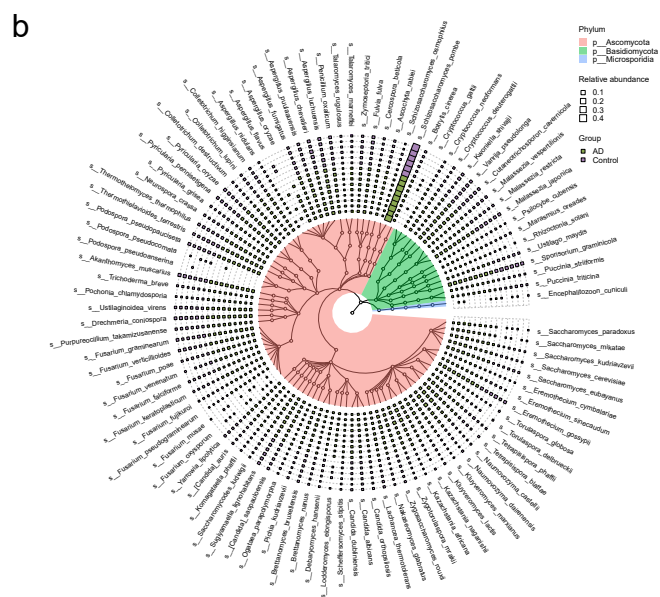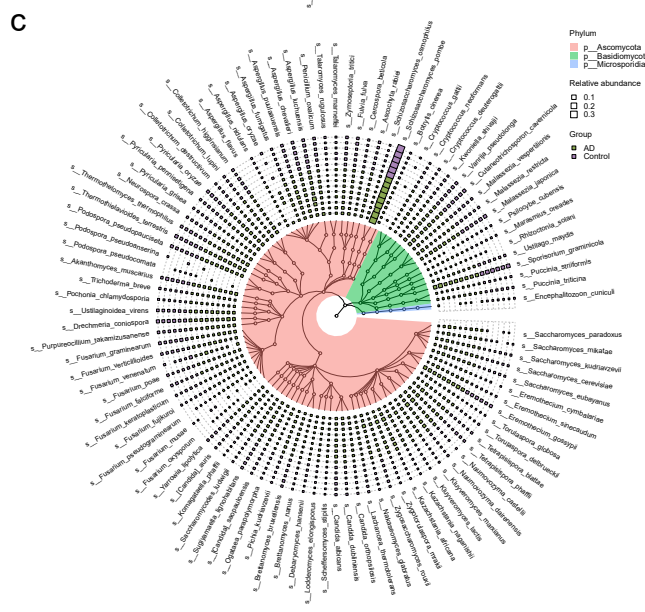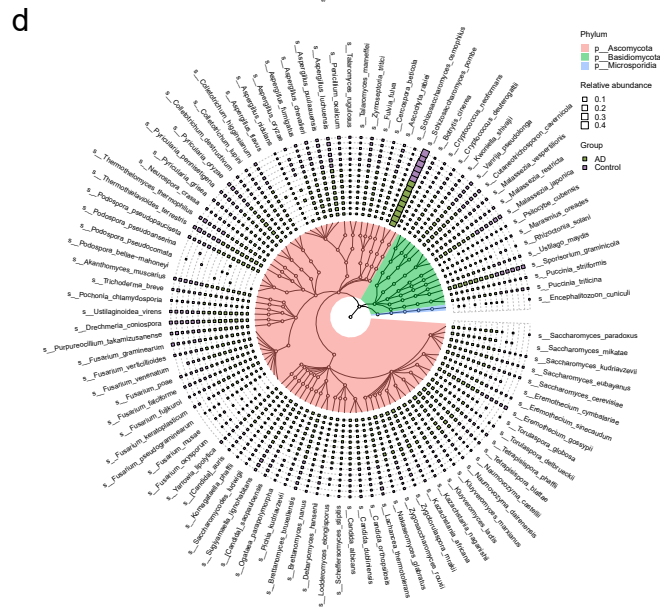

Supplement: Supplementary file 1 [file ijms-25-11472-s001.zip › Supplementary Figure S2.pdf]

a

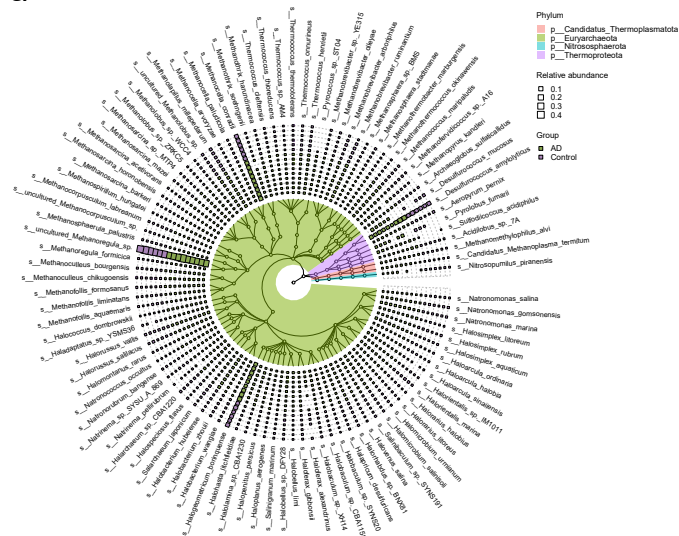

b

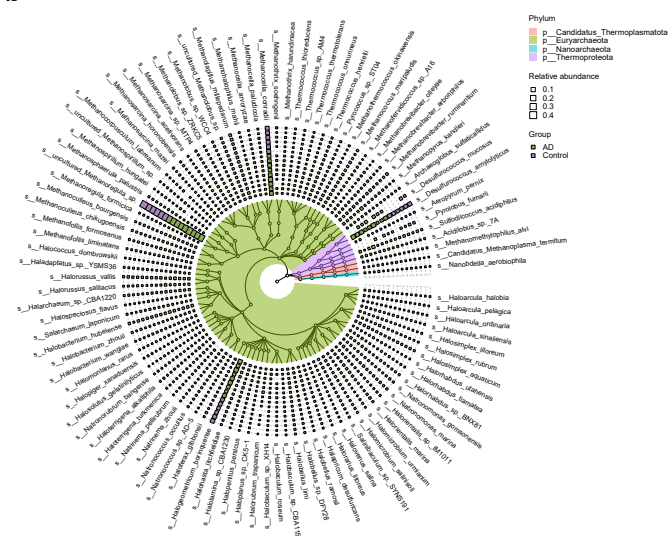

c

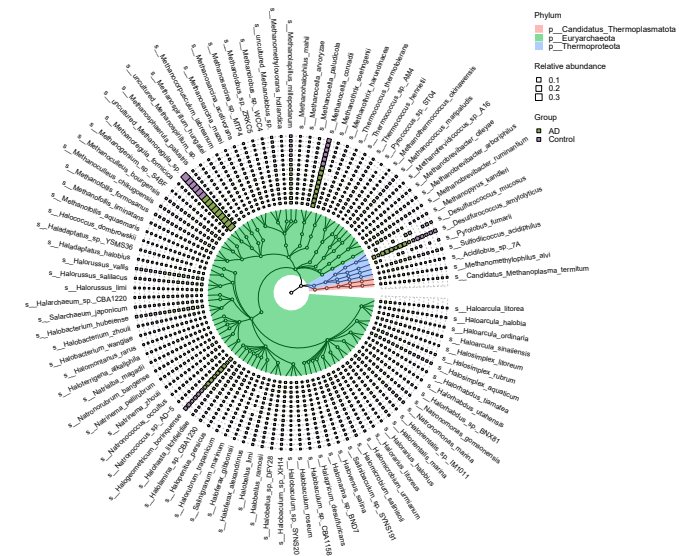

d

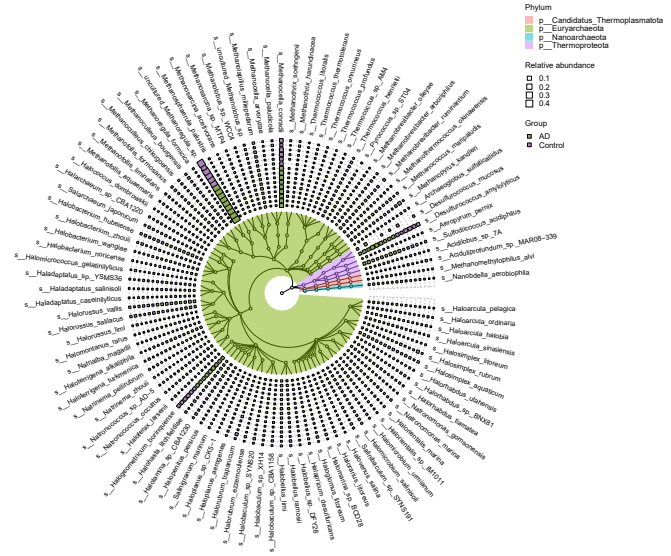

Supplement: Supplementary file 1 [file ijms-25-11472-s001.zip › Supplementary Figure S3.pdf]

a

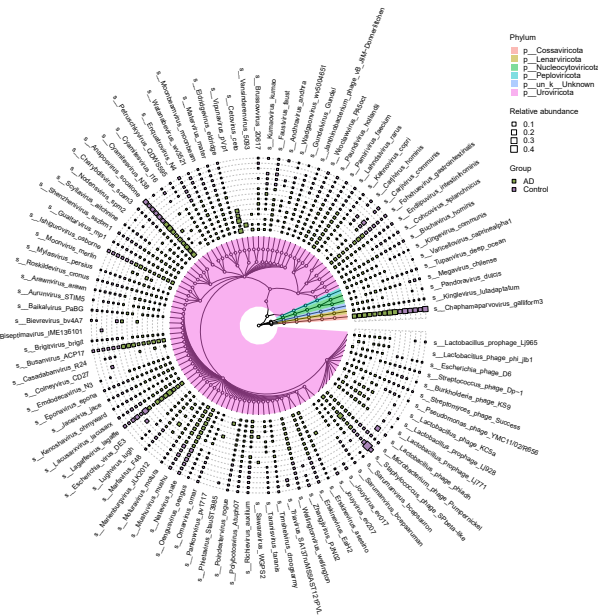

b

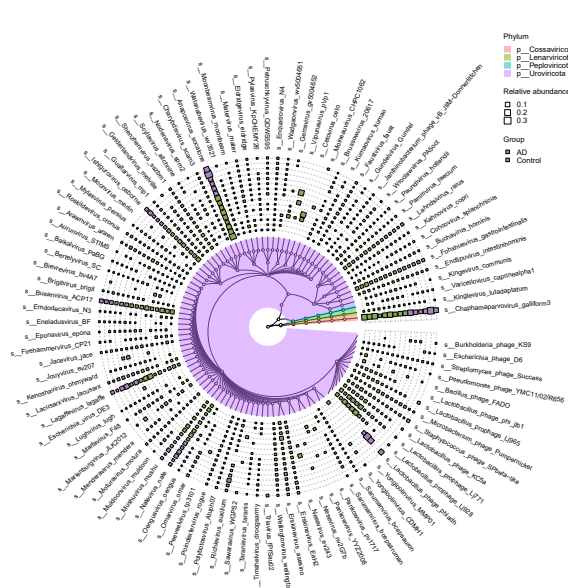

c

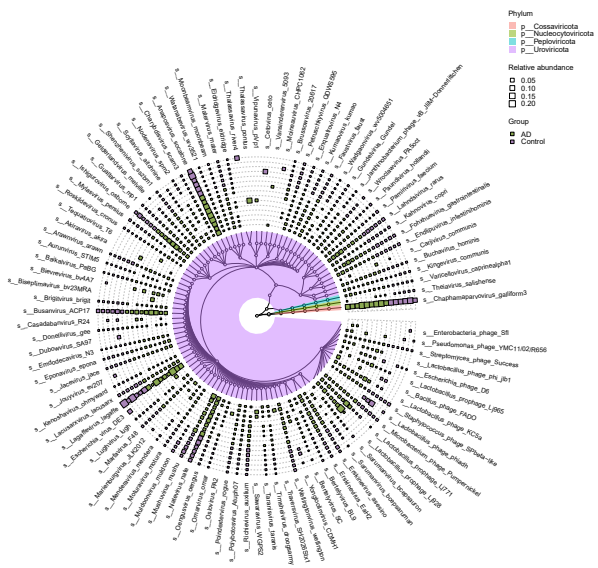

d

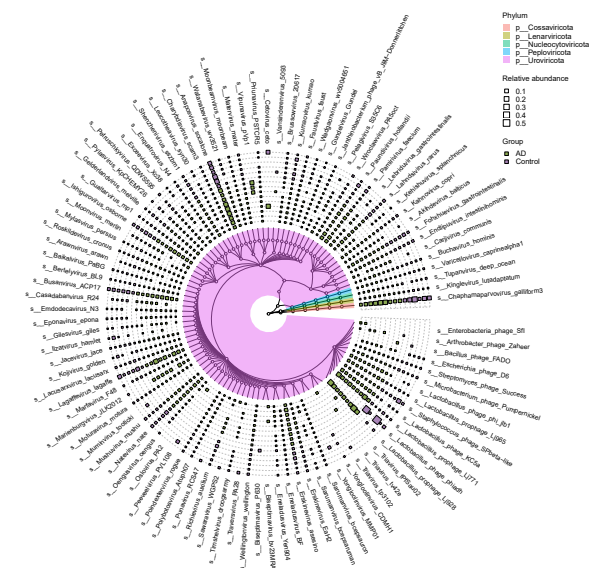

Supplement: Supplementary file 1 [file ijms-25-11472-s001.zip › Supplementary Figure S4.pdf]

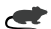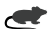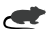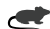

3

4

5

6

Age (months)

## Bacteria

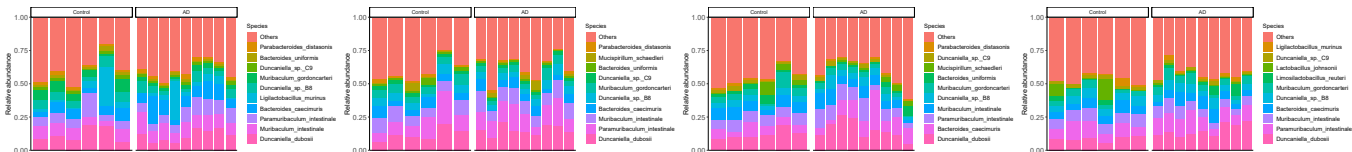

## Fungi

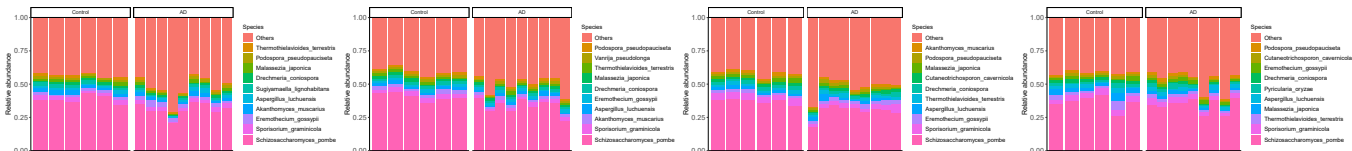

## Archaea

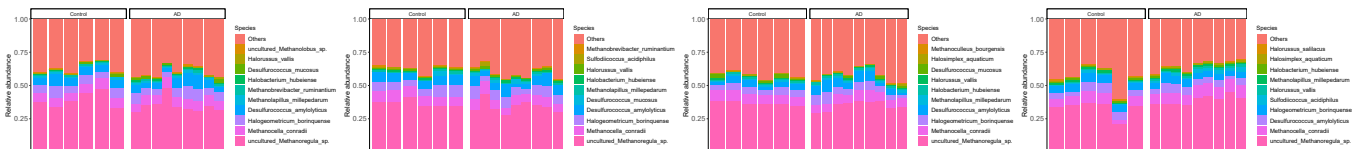

## Viruses

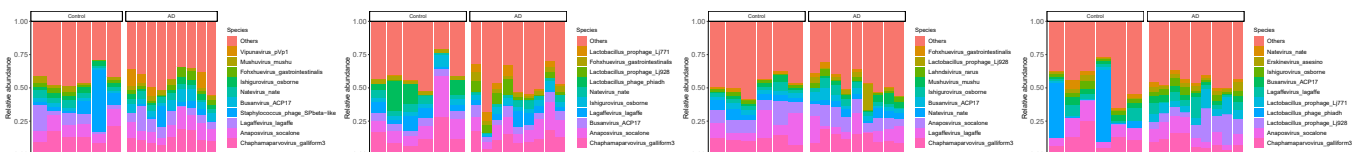

Supplement: Supplementary file 1 [file ijms-25-11472-s001.zip › Supplementary Figure S5.pdf]

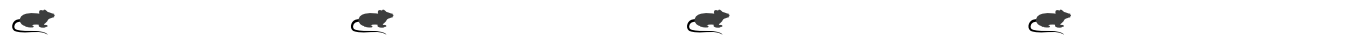

3

4

5

6

Age (months)

## Bacteria

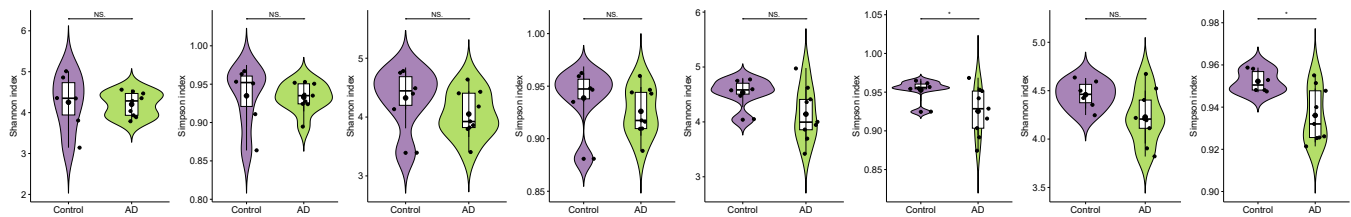

## Fungi

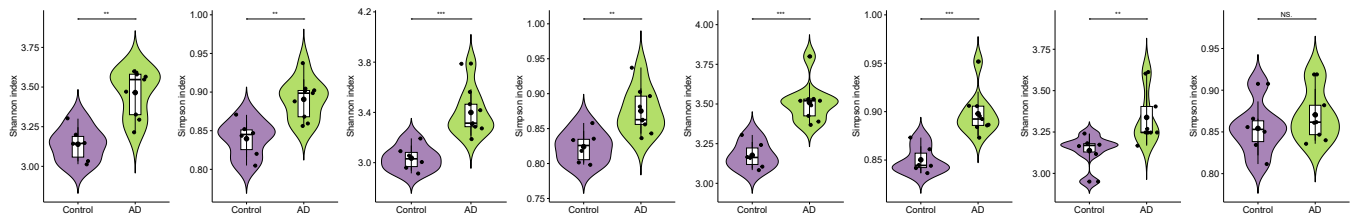

## Archaea

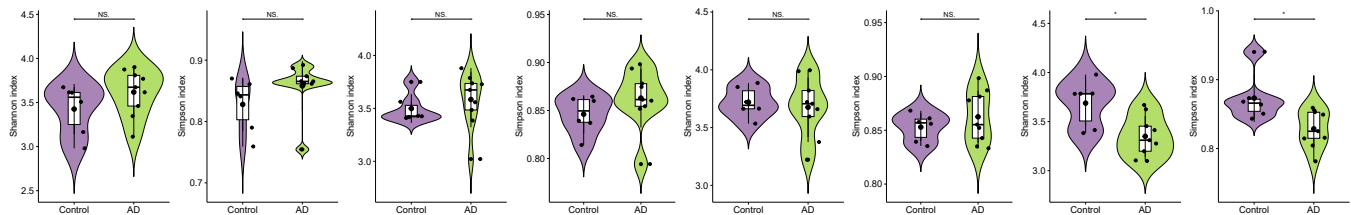

## Viruses

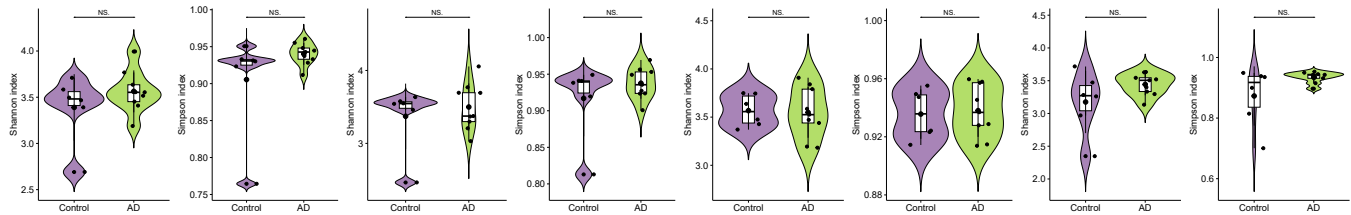

Supplement: Supplementary file 1 [file ijms-25-11472-s001.zip › Supplementary Figure S6.pdf]

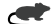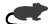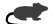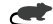

3

4

5

6

Age (months)

MetaCyc

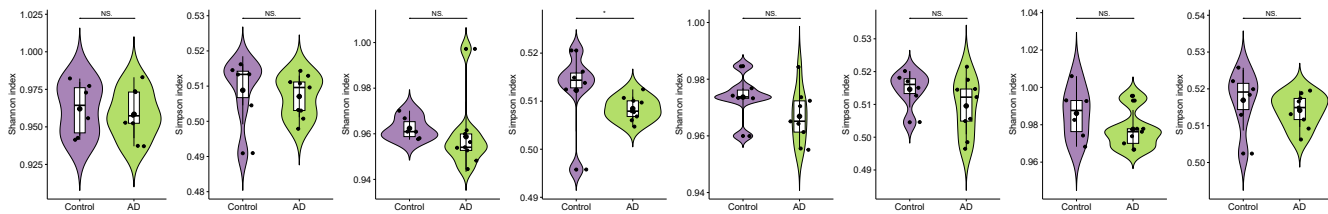

KO

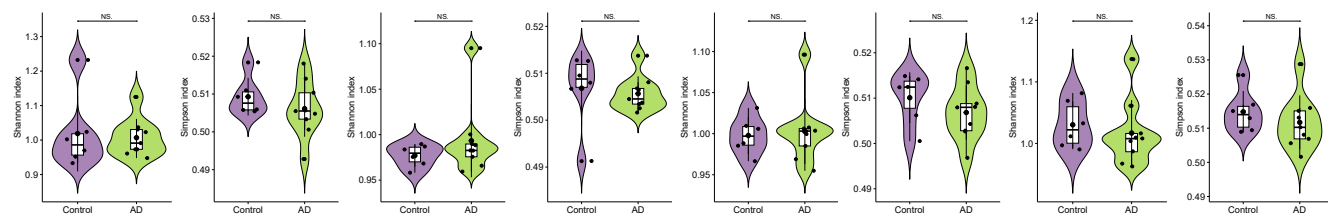

GO

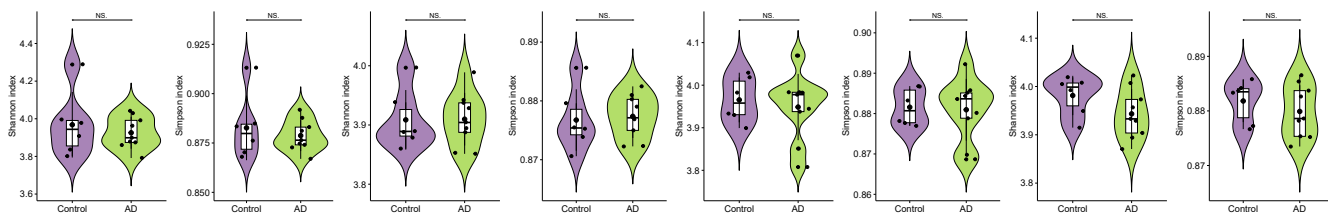

eggNOG

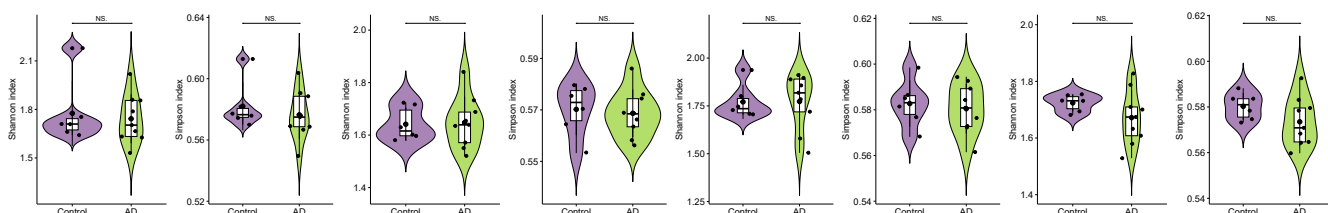

Supplement: Supplementary file 1 [file ijms-25-11472-s001.zip › Supplementary Figure S7.pdf]

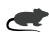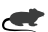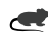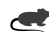

3

4

5

6

Age (months)

Control

AD

MetaCyc

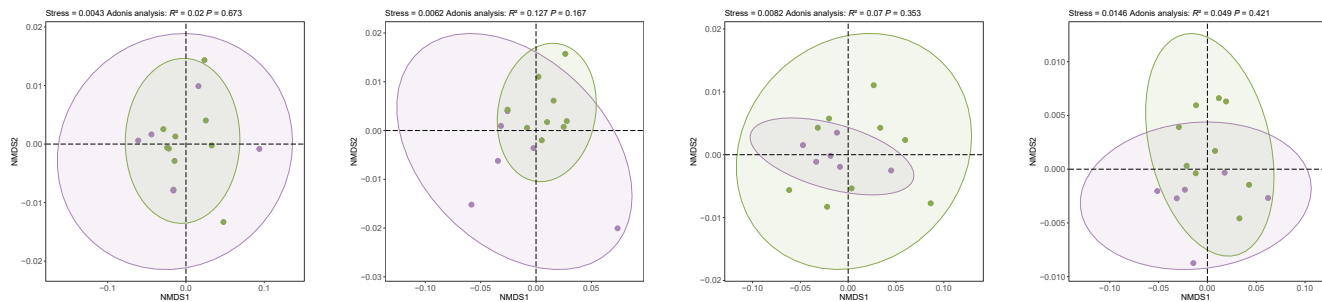

KO

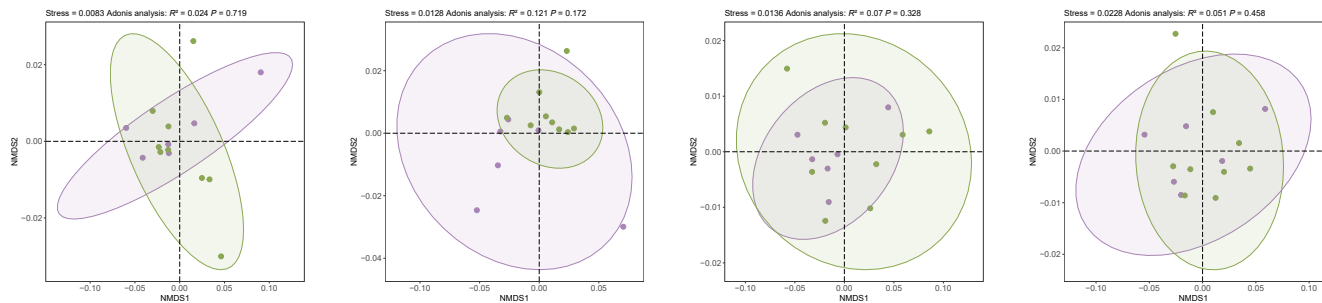

GO

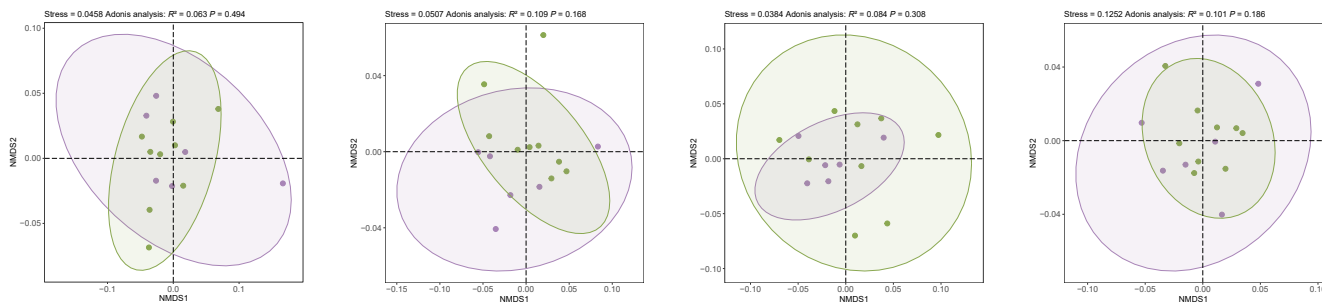

eggNOG

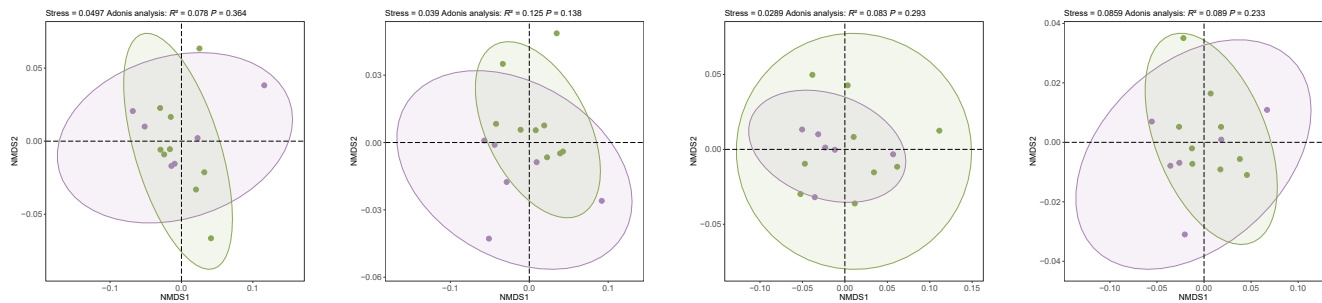

Supplement: Supplementary file 1 [file ijms-25-11472-s001.zip › Supplementary Figure S8.pdf]

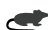

3

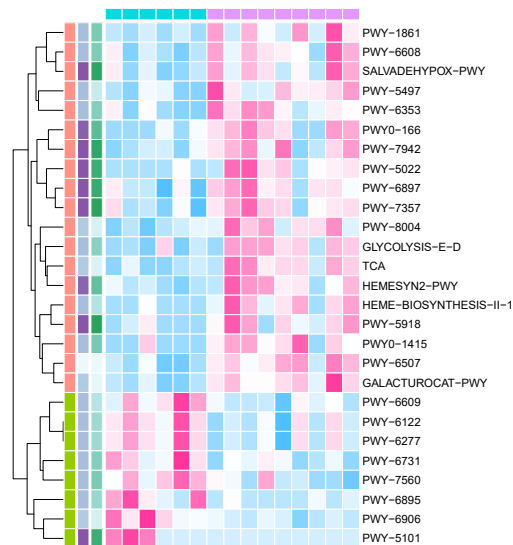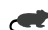

4

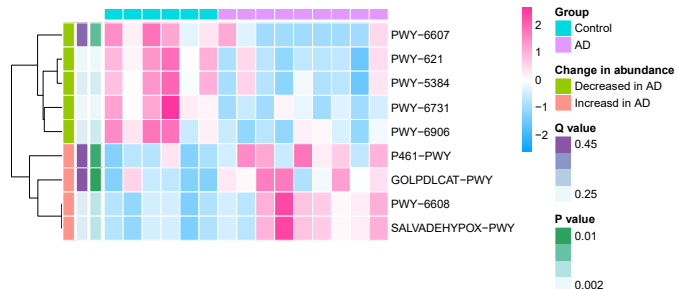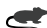

5

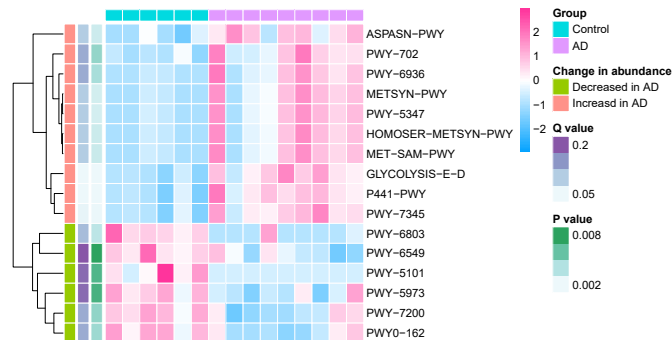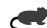

6

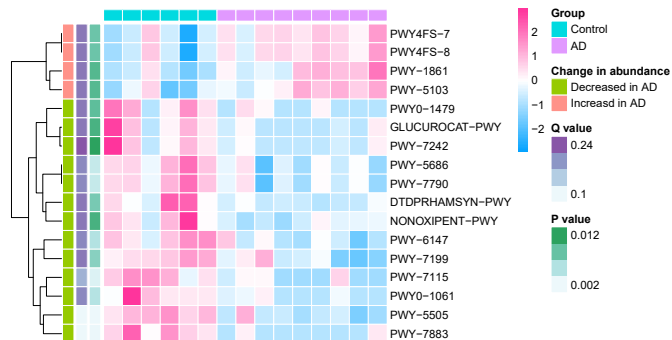

Supplement: Supplementary file 1 [file ijms-25-11472-s001.zip › Supplementary Figure S9.pdf]
